# Granule Associated Serine Proteases of Hematopoietic Cells – An Analysis of Their Appearance and Diversification during Vertebrate Evolution

**DOI:** 10.1371/journal.pone.0143091

**Published:** 2015-11-16

**Authors:** Srinivas Akula, Michael Thorpe, Vamsi Boinapally, Lars Hellman

**Affiliations:** Department of Cell and Molecular Biology, Uppsala University, Uppsala, The Biomedical Center, Box 596, SE-751 24, Uppsala, Sweden; University of Gdansk, POLAND

## Abstract

Serine proteases are among the most abundant granule constituents of several hematopoietic cell lineages including mast cells, neutrophils, cytotoxic T cells and NK cells. These proteases are stored in their active form in the cytoplasmic granules and in mammals are encoded from four different chromosomal loci: the chymase locus, the met-ase locus, the T cell tryptase and the mast cell tryptase locus. In order to study their appearance during vertebrate evolution we have performed a bioinformatic analysis of related genes and gene loci from a large panel of metazoan animals from sea urchins to placental mammals for three of these loci: the chymase, met-ase and granzyme A/K loci. Genes related to mammalian granzymes A and K were the most well conserved and could be traced as far back to cartilaginous fish. Here, the granzyme A and K genes were found in essentially the same chromosomal location from sharks to humans. However in sharks, no genes clearly identifiable as members of the chymase or met-ase loci were found. A selection of these genes seemed to appear with bony fish, but sometimes in other loci. Genes related to mammalian met-ase locus genes were found in bony fish. Here, the most well conserved member was complement factor D. However, genes distantly related to the neutrophil proteases were also identified in this locus in several bony fish species, indicating that this locus is also old and appeared at the base of bony fish. In fish, a few of the chymase locus-related genes were found in a locus with bordering genes other than the mammalian chymase locus and some were found in the fish met-ase locus. This indicates that a convergent evolution rather than divergent evolution has resulted in chymase locus-related genes in bony fish.

## Introduction

Approximately 560 protease genes are present in primate genomes and around 150 of these encode proteases of the serine protease class. Slightly more than 50% of these belong to the trypsin/chymotrypsin related serine protease family making them one of the major protease families [1]. Serine proteases of this latter family are found at very high levels in the granules of cells of several hematopoietic cell lineages including mast cells, neutrophils, cytotoxic T cells and NK cells, where they can account for up to 35% of the total cellular protein [2]. Members of this protease family take part in a large number of physiological processes including blood coagulation, clot resolution, complement activation, food digestion, fertilization, blood pressure regulation, tissue homeostasis, and immunity. Members of this gene family include thrombin, plasmin, tissue plasminogen activator (TPA), urokinase, coagulation factors VII, IX, X, XI, XII and protein C, complement components B, D, C2, C1r, C1s, factor I, the numerous tissue kallikreins, acrosin, leydin, testin, pancreatic trypsin, chymotrypsin, elastase, haptoglobin and all the different granule associated hematopoietic serine proteases that are the theme of this communication.

In mast cells we find the chymase, a chymotryptic enzyme as well as the tryptase, a tryptic enzyme and also granzyme B [3, 4]. This latter enzyme is an asp-ase and has a primary specificity for negatively charged amino acids [5, 6]. Human neutrophils express four active proteases: neutrophil elastase (N-elastase), proteinase 3, cathepsin G and neutrophil serine protease 4 (NSP-4) [4, 7, 8]. Human neutrophils also express a related proteolytically inactive, antibacterial protein named CAP 37 or azurocidin [9]. Of the neutrophil proteases cathepsin G also seems to be expressed in human mast cells but not in rat peritoneal mast cells or in mouse bone marrow derived mast cells (BMMCs) and not in most mouse mast cell lines [10–12]. Cytotoxic T cells express granzymes A, K, B and H and NK cells express granzymes M and A but not B [13, 14]. All of these proteases are located in four chromosomal loci in humans: the chymase locus, the met-ase locus, the T cell tryptase and the mast cell tryptase locus [4]. In humans, the chymase locus contains the mast cell chymase, neutrophil cathepsin G, and the T cell granzymes B and H [15, 16]. The human met-ase locus contains granzyme M, proteinase 3, N-elastase, azurocidin, NSP-4 and complement factor D (CFD) [4]. NSP-4 is encoded from the gene PRSS57 [8]. The human granzyme A/K locus, also named the T cell tryptase locus, only contains two proteases, granzymes A and K, whereas the mast cell tryptase locus is the most complicated, having a number of secreted and membrane bound trypsin-related enzymes in two separate regions of the locus [4, 17]. The mast cell tryptases are distantly related to the other three loci and are therefore not included in this study. However, we have recently performed an analysis of this locus in mammals and seen that it has essentially the same basic structure from marsupials to placental mammals, and probably a similar locus in monotremes [17]. The other three loci are more closely related and may have appeared by two whole genome duplications during early vertebrate evolution [4].

The chymase locus has previously been shown to have diversified quite extensively during mammalian evolution. The rat locus is 15 times larger than the corresponding locus in dogs and the number of genes has increased from 4 functional genes in humans and dogs to 28 in rats [18]. The mouse locus is only 2.5 times larger than the human locus but contains 15 functional genes [4, 18]. Interestingly, additional subfamilies have also appeared in some branches of the placental family tree. Rodents have one extra subfamily of chymases named β-chymases, which have no obvious members in other placental mammalian lineages with one potential exception in dogs and a potential additional one highlighted here in cats. Dogs have an additional chymase gene, which at the protein level is more similar to the α-chymase but at the nucleotide level is more related to the rodent β-chymases [15]. In ruminants, exemplified by cattle and sheep, an additional subfamily of related proteases, the duodenases, are found in the chymase locus [16]. These new members are expressed in the intestinal region and not in hematopoietic cells, and therefore have been named duodenases due to their expression pattern [19, 20]. Specificity analyses indicates dual tryptase and chymase activities of these enzymes [21].

As alluded to above, we have a relatively clear picture of the genes for these proteases and their expression patterns in mammals, however, there has been very few, if any, studies of their presence in non-mammalian vertebrate species. In order to close this gap in our understanding of the appearance and diversification of this complex family of granule proteins, here we present detailed bioinformatic analyses of three of these four loci in a panel of metazoan animals spanning from sea urchins and tunicates to placental mammals.

## Materials and Methods

### Gene Loci

We used the National center for Biotechnology Information (NCBI) (http://www.ncbi.nlm.nih.gov/) and the Ensemble (http://www.enseml.or/index.html) databases for identifying the gene loci and the individual genes for the different hematopoietic serine proteases. Human and mouse hematopoietic serine protease protein sequences were initially used as query sequences in translation BLAST, Translate Basic Local Alignment Search Tool (TBLASTN) in the nucleotide collection (nr/nt) database. The identified genes in fish, frogs, reptiles and birds were then used to screen for potential additional homologous genes to complete the picture. The gene loci were compiled from the resulting data and the sizes of the genes, including the distance between the neighboring genes were calculated and used to construct in scale maps of the loci. The figures were made in Adobe Illustrator (CS6). The Gene bank accession numbers used in constructing the figures are listed in the supporting information (S1 file)

### Alignment and Phylogenic Analyses

Alignments: The selected serine proteases (368) were aligned in version 7 of MAFFT (http://mafft.cbrc.jp/alignment/server/), using with BLOSUM62 as the scoring matrix and as a option G-INS_I strategy for optimal results for sequences with global alignment, with default parameters [22]. To check the alignment conservation and confidence the GUIDANCE2 server (http://guidance.tau.ac.il/ver2/) was used [23]. To verify the multiple sequence alignment from MAFFT, another alignment algorithm (T-coffee) was used to verify that both alignments were similar.

Phylogenic analyses: For all 368 proteases, the entire sequence of the active form, not including the signal sequence and activation peptide were used in the multiple alignments. The phylogenetic analyses were performed using a Bayesian approach as implemented in MrBayes version 3.1.2. Markov Chain Monte Carlo (MCMC) analyses were used to approximate the posterior probabilities of the trees. Analyses were run using the MrBayes manual standard protocol [24]. The phylogenetic trees were drawn in FigTree 1.4.2 (http://tree.bio.ed.ac.uk/software/figtree/). The topologies of all Bayesian phylogenetic trees supported by posterior probabilities (PP) were verified with Bootstrap ML and distance trees using PHYLIP program package.

The PHYLIP package (v3.69)(http://evolution.genetics.washington.edu/phylip.html) was used for constructing maximum-likelihood and distance trees [25]. For the distance method PROTDIST and FITCH (JTT matrix model without using an out-group species) were used. For the bootstrap analyses SEQBOOT, PROTDIST, NEIGHBOR and CONSENSE were used to generate 100 replicate data sets from the PHYLIP package. The phylogenetic tree was drawn in Fig Tree (v1.4.2) http://tree.bio.ed.ac.uk/software/figtree/) (S1 Fig). The maximum likelihood tree was generated by SEQBOOT, PROML and CONSENSE to generate 100 replicates. The phylogenetic tree was drawn in Fig Tree (1.4.2) http://tree.bio.ed.ac.uk/software/figtree/) (S2 Fig).

## Results

**Fig 1:**
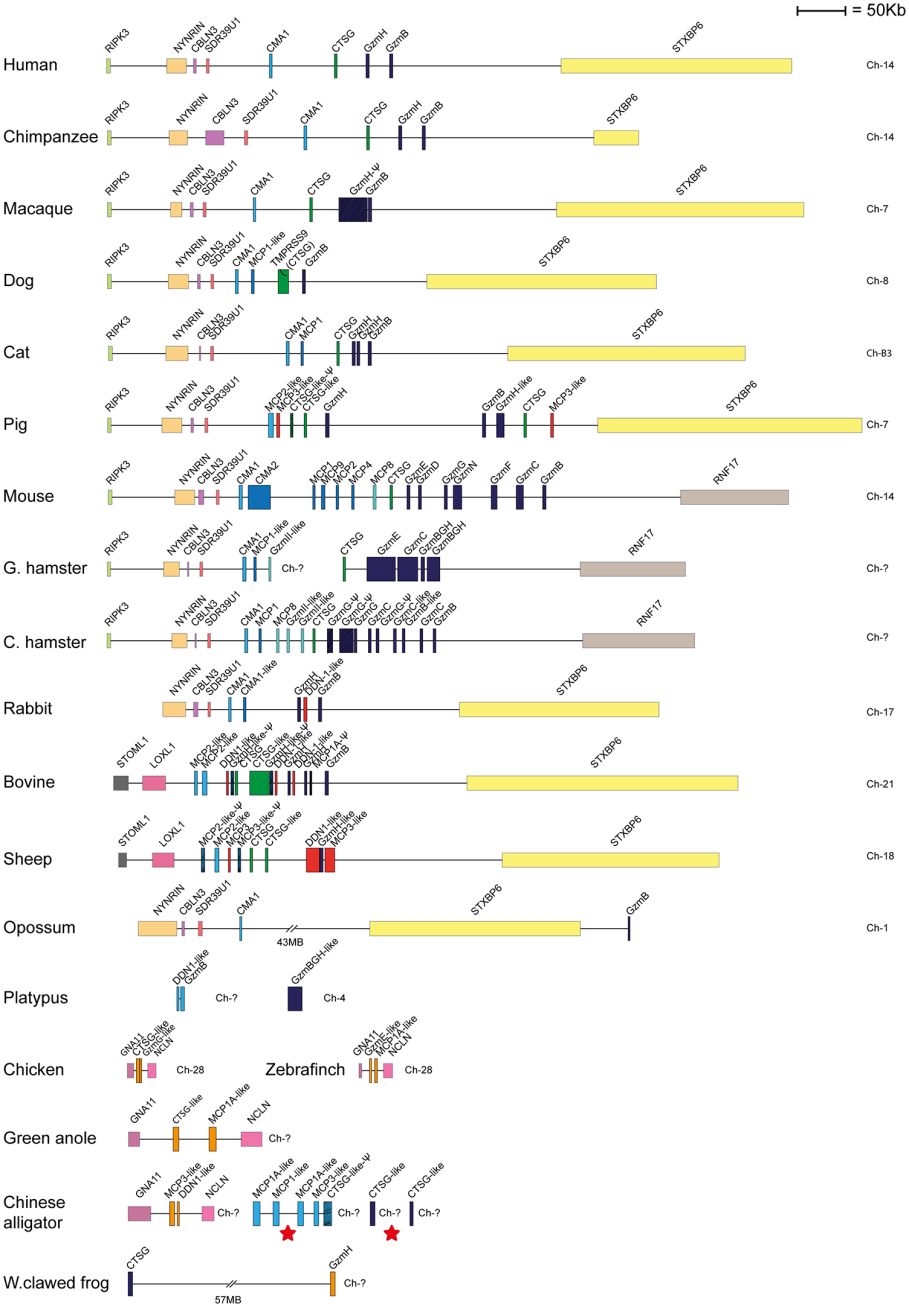
The chymase locus. The genes encoding serine proteases are depicted in double height to easily locate them in the maps. The bordering genes are included to trace the origin of the locus and to also define changes that have occurred upstream or downstream of the locus. Gene names for the serine proteases, as given in the database, particularly in fish and amphibians do not match their closest homologs in mammals, and can sometimes be quite misleading. The α-chymase related genes are depicted in light blue, the β-chymases in slightly darker blue, cathepsin G in green, the M8 family in a darker green, the granzymes in dark blue and the duodenases in red. The Chinese alligator locus and the two individual contigs that appears most closely related to the mammalian chymase locus genes are marked with red stars and the genes are depicted in light or dark blue. One of the *Xenopus* genes that also cluster with these genes (Fig 6) are also shown in dark blue. The genes from the other loci in reptiles, birds and frogs that are more distantly related to the mammalian chymase locus genes are shown in light brown.

**Fig 2:**
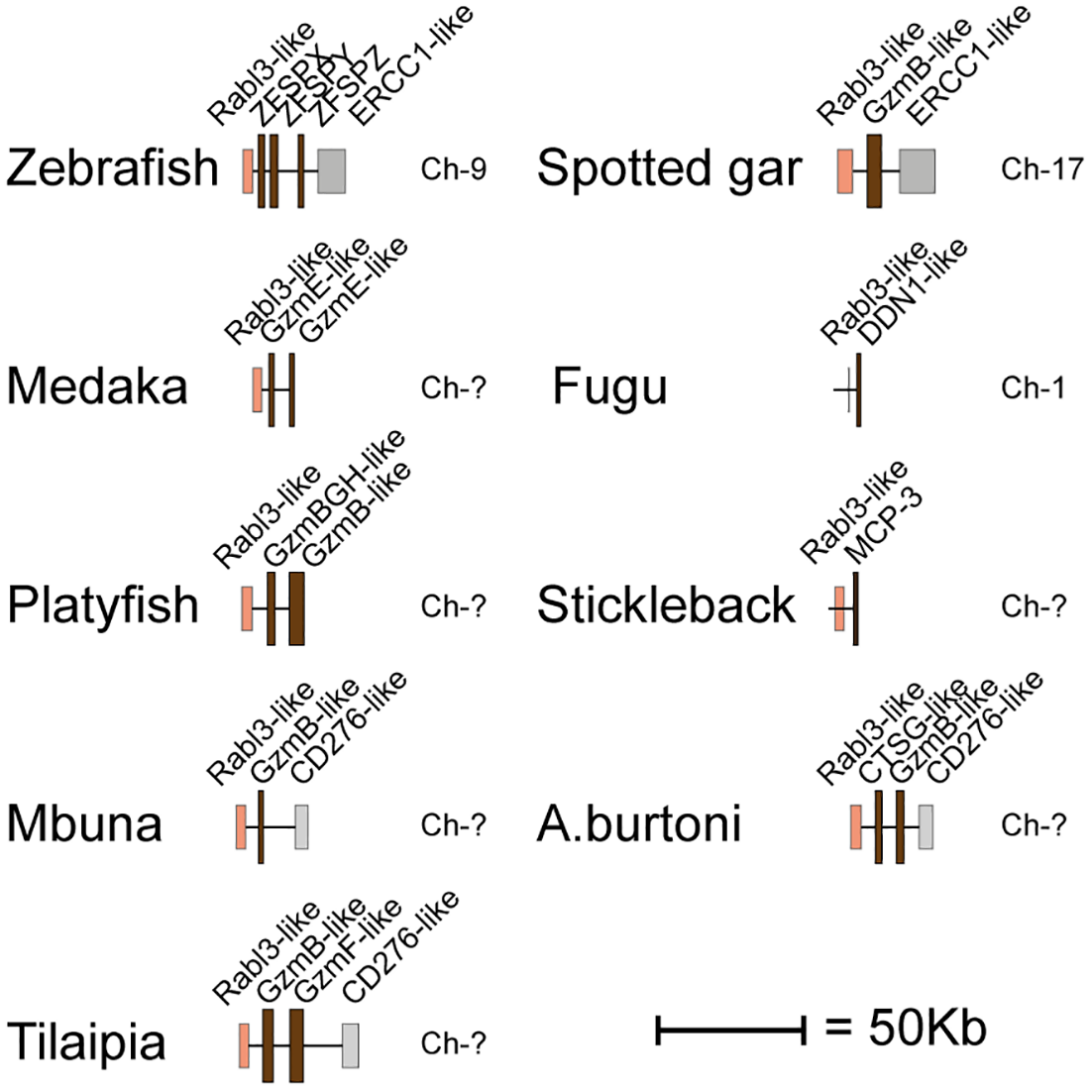
The fish chymase locus. The locus that harbors chymase-related genes in fish and do not show homology to neither the chymase locus nor the met-ase locus of mammals. The genes encoding serine proteases are depicted in double height to easily locate them in the maps. The different fish chymase locus-related genes are shown in dark brown.

**Fig 3:**
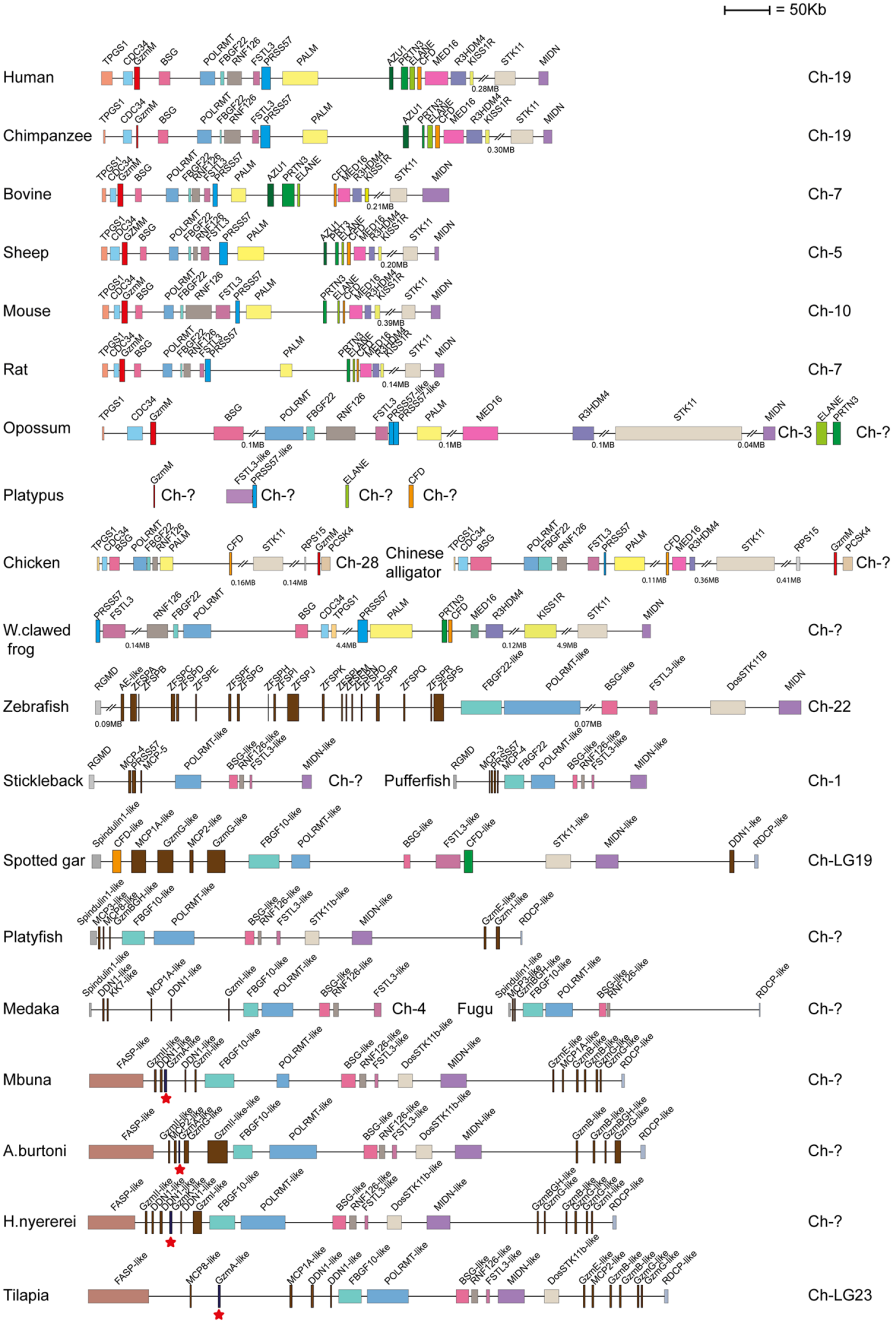
The met-ase locus. A. The genes encoding serine proteases are depicted in double height to easily locate them in the maps. The bordering genes are included to trace the origin of the locus and to also define changes that have occurred upstream or downstream of the locus. Gene names, as given in the database, particularly in fish and amphibians do not match their closest homologs in mammals, and can sometimes be quite misleading. CFD is depicted in orange, granzyme M in red, PRSS57 in blue, N-elastase, azurocidin and proteinase 3 in various shades of green. The fish chymase locus-related genes in the met-ase locus are shown in dark brown. The granzyme A/K genes located in the cichlid met-ase locus are marked by red stars.

**Fig 4:**
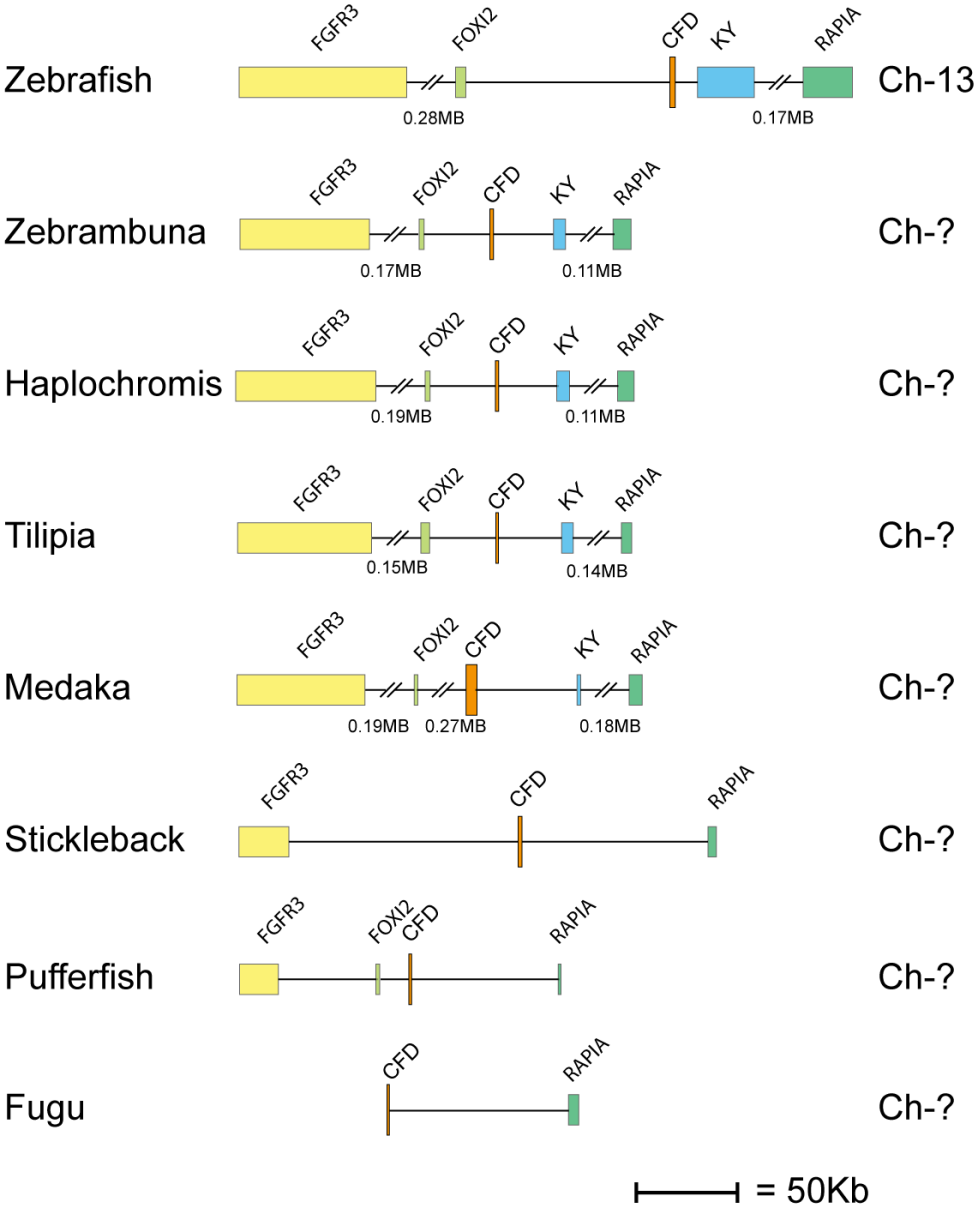
The second locus encoding complement factor D in fish. The genes encoding serine proteases are depicted in double height to easily locate them in the maps. This locus encodes only CFD in most fish species. The only exception so far is in the spotted gar, which has one copy in the met-ase locus (Fig 3). The CFD gene is depicted in orange.

**Fig 5:**
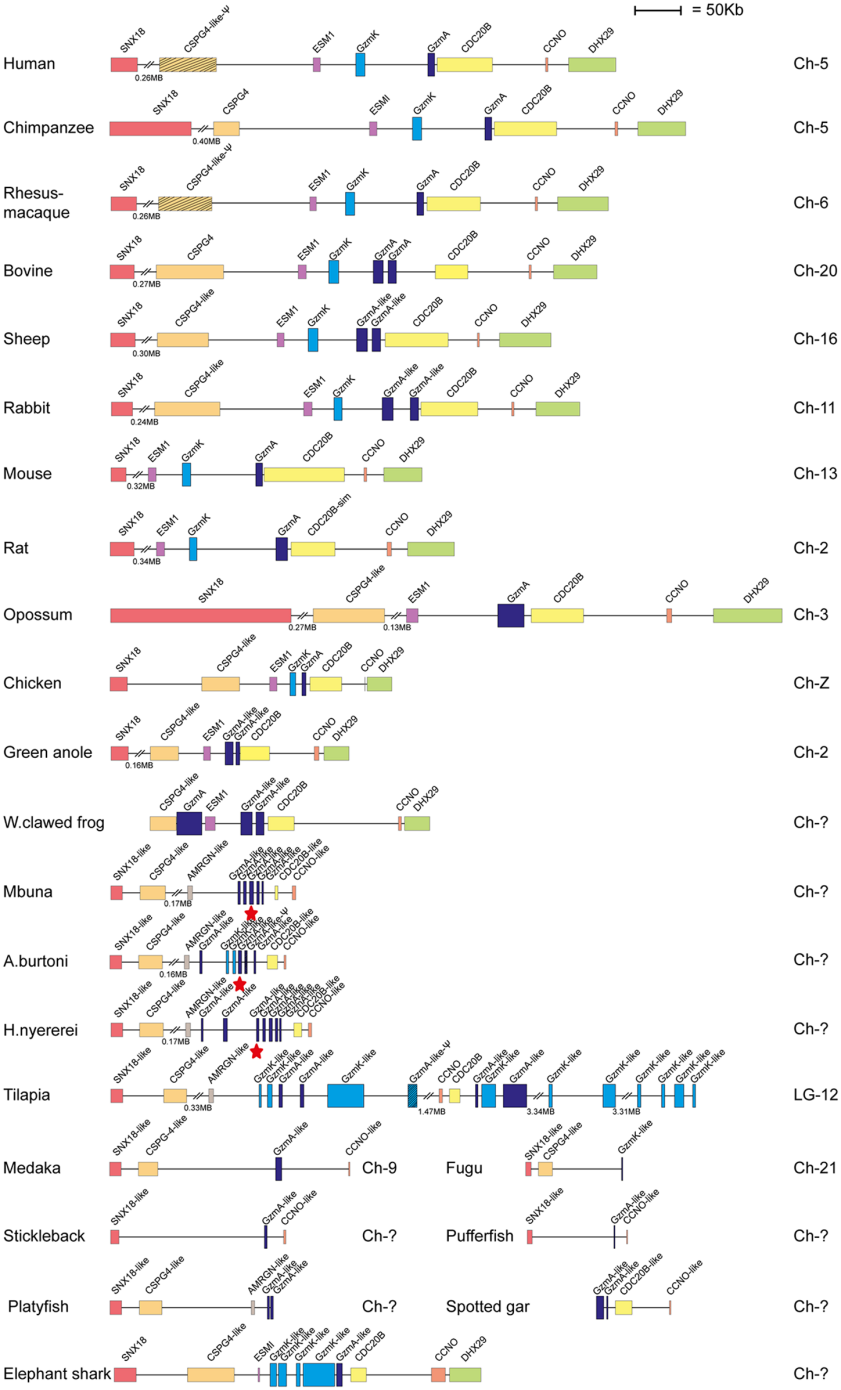
The granzyme A/K locus. The genes encoding serine proteases are depicted in double height to easily locate them in the maps. The bordering genes are included to easily trace the origin of the locus and to also define changes that have occurred upstream or downstream of the locus. Gene names in the database as A or K in fish and amphibians do not always match their closest homologs in mammals. The granzyme A and K genes from mammals, birds and reptiles form distinct sub-branches and are therefore depicted in dark and light blue respectively. The color coding in fish is not so distinct but the genes have been coded light and dark blue as named in the database. However, as seen in Fig 6E, they cluster together and not within the mammalian sub-branches for A and K. Three genes of the cichlid GzmA/Ks that have specificity triplets as the chymases, SGG, have been marked by red stars.

Human and mouse hematopoietic serine protease sequences were used as query sequences to identify similar sequences in a large panel of vertebrate genomes in the NCBI database using the TBLASTN algorithm. The ensemble database was also later screened for related sequences to obtain the best coverage of the various genomes included in this study. The different gene loci were identified, the size of the genes and the distances between genes were calculated and used to produce maps drawn to scale of these chromosomal regions as presented in Figs 1, 2, 3, 4 and 5. The chymase locus is shown in Fig 1, the separate locus encoding chymase locus-related genes in fish in Fig 2, the met-ase locus in Fig 3, a separate locus carrying a CFD gene in most fish species except the gar in Fig 4 and the T cell tryptase locus (granzyme A/K locus) in Fig 5. In order to try and identify the early ancestors of these loci, a few additional species were also analyzed, including tunicates, sea urchins and lampreys.

### The Chymase Locus

In all primates that were analyzed, the chymase loci had a very similar structure. The locus was bordered at one end by the mast cell α-chymase (Cma1) and at the other end by granzyme B (Gzm B) (Fig 1). In the middle of the locus cathepsin G (CTSG) and one additional gene, granzyme H (Gzm H), was found. The same number of functional genes was also previously found in dogs. However previously, the dog locus also harbored a pseudogene closely related to the Cma1 gene. This gene has suffered a single nucleotide deletion resulting in a frame shift within exon 4, rendering it inactive [15]. A recent update of the dog genome has resulted in major changes to this view. The dog granzyme H is now no longer present and the pseudogene is rendered functional. Therefore the locus now looks quite different from what we and others have previously published [4, 15, 18]. The locus now contains two chymases: one α-chymase (Cma1) and one that is more closely related to the β-chymases (Cma2). The locus also contains a cathepsin G and a granzyme B gene (Fig 1). We have previously tried to clone the pseudogene (now the β-chymase, Cma2) by PCR amplification with specific primers from different dog tissues without success, which may indicate that it is not being expressed [4]. In rodents (mice and rats) a massive expansion of the locus is seen where two new subfamilies of proteases, the β-chymases, which are closely related to Cma1, and the mMCP-8 subfamily that is more closely related to the granzymes and cathepsin G are observed. Both of these latter subfamilies have previously only been found in rodents, with the possible exception of the β-chymase like gene in dogs [15]. Interestingly, a β-chymase- like gene was also found in the cat genome at approximately the same position as the dog and rodent genes indicating that β chymases are relatively old, possibly appearing at the base of placental mammals, and that they have been lost in some lineages such as the primates. In ungulates (hooved mammals), as represented by cattle, sheep and pigs, the locus also has a novel subfamily named duodenases. This subfamily of proteases has changed tissue specificity and is now expressed in the duodenum where they most likely participate in food digestion [19, 20]. The number and order of the genes in the cattle locus has also changed quite dramatically for every update of the genome sequence. The locus now looks quite different from what has been published previously [16]. There are now three duodenase genes and they are found on either side of the central cathepsin G gene. In cattle, this locus is also bordered by an α-chymase and granzyme B gene, similar to what was seen in primates, rodents and dogs (Fig 1). Sheep and cattle are the only two species, so far, where there have been recent duplications of both the α-chymase gene and cathepsin G (Fig 1). The genes are very similar, 93–94% identity, indicating that these duplications occurred quite recently. However, this was most likely before the split of the lineages leading to sheep and cattle, which has been estimated to be sometime between 20 and 40 million years ago [16]. Looking in marsupials, exemplified by the American opossum, the chymase locus was broken up into two parts by an inversion (Fig 1). This conclusion is based on the observation that the position of one of the flanking genes (STXBP6) is in a reverse orientation (Fig 1) [16]. Only two of the classical chymase locus genes, Cma1 and Gzm B, have been identified in the opossum genome. In the monotremes, another non-placental mammalian lineage, a partial genome sequence is available for the Australian platypus. Here, three genes on two different contigs with close homology to the placental chymase locus genes were found: one harboring two genes, and one contig with only one chymase locus-related gene. In marsupials and most placental mammals this locus was bordered at one end by the genes RIPK3, NYNRIN, CBLN3 and SDR39U1, and at the other end by STXBP6. In sheep and cattle, the bordering genes at one end where NYNRIN was found has been affected by a rearrangement and instead was now bordered by the STOML1 and LOXL1 genes. The NYNRIN gene is now found on another chromosome (Chr10 in cattle and Chr7 in sheep). In mice, the opposing end of the locus had undergone rearrangement, replacing STXBP6 with RNF17 as a bordering gene (Fig 1). In birds there were no protease genes in the region close to these bordering genes, however related genes were found in another chromosomal region (Fig 1). Two distantly related genes, cathepsin G-like (CTSG-like) and granzyme G-like (GzmG-like) were found in chickens and also in both zebra finches and the green anole lizard. However, in Chinese alligators, where the genome is still not complete, 9 related genes on four different contigs were found. One contig was similar to the genes from birds and the green anole (Fig 1). Interestingly, one of the other larger and two of the very small contigs were more closely related to the classical chymase locus genes in mammals (shown later in the phylogenetic analysis) and may actually represent the true orthologs of the mammalian locus. This locus and the two short contigs are marked by red stars in Fig 1. A third locus with chymase locus related genes was also observed in this species (Fig 1). In amphibians, as represented by *Xenopus tropicalis* or Western clawed frog, two related genes named CTSG and GzmH in the genome assembly were found. They were in the same contig but separated by more than 115 Mb. These two genes may actually represent members of both types of loci found in the Chinese alligator with the CTSG gene found in the classical chymase locus and GzmH within the bird chymase-like locus. It should also be made clear that the names in birds, reptiles and amphibians do not always correctly match the corresponding genes in mammals. The homology is sometimes too low to say which gene of the mammalian chymase locus genes that is the closest homolog as the names becomes somewhat random in the genome assemblages.

No genes similar to the chymase locus genes were detected in cartilaginous fish, lampreys, tunicates or sea urchins. However, genes distantly related to the chymase locus genes of mammals were observed in bony fish (Fig 2). Here, the bordering genes were different from both the ones found in mammals, as well as reptiles and birds, indicating a third chromosomal region. Between one to three related genes in various fish species were found to be located in a region between the bordering genes rabl3-like and CD276 or ERCC-1 like: ERCC-1 like in the zebrafish and spotted gar, and CD276 in the cichlids (zebra mbuna, haplochromis and Nile tilapia) and platyfish. However, chymase locus-related genes were also found in the met-ase and the granzyme A/K loci of bony fish, as described in the following sections, indicating a convergent evolution of genes duplicated in these loci, resulting in genes with structural and potentially functional similarity to the chymase locus genes of mammals.

### The Met-Ase Locus

In primates, the met-ase locus encodes a number of relatively distantly related serine protease genes: Gzm M at one end, PRSS57 located centrally, and several of the other neutrophil proteases and CFD (also named adipsin) at the other end (Fig 3). The neutrophil proteases found in this locus are proteinase 3, N-elastase and a closely related but enzymatically inactive protein named azurocidin. The latter was only found in some placental mammals but not in rodents, marsupials or other tetrapods and not in fish (Fig 3). The PRSS57 gene also encodes a neutrophil expressed protease named NSP-4, a recently identified low abundant tryptic enzyme found in neutrophil granules together with the other neutrophil proteases [8]. In cattle there was a duplication of the CFD gene although no other major changes were seen. Mice and rats also had very similar met-ase loci compared to the primates except for the lack of the gene encoding azurocidin (Fig 3). The situation in opossum was slightly more difficult to resolve as the region covering Gzm M and PRSS57 was similar in organization but had expanded in size, where the remaining part of the locus was missing, with N-elastase and proteinase 3 genes on a small unassigned contig (Fig 3). In chickens, only CFD and Gzm M were found whereas Chinese alligators also had the gene for PRSS57 (Fig 3). Interestingly, when looking at amphibians both the African and Western clawed frogs, (*Xenopus laevis* or *tropicalis*) had lost the gene for Gzm M but instead had two copies of the proteinase 3 gene and one gene for PRSS57. In addition, no genes for N-elastase or azurocidin were identified. In the various fish species there was only one clear homolog to any of the genes found in mammals, the CFD gene, which was clearly identifiable in the met-ase locus of the spotted gar. In other fish species CFD was located in another chromosomal region (Fig 4). The spotted gar had two copies of the CFD gene located in different parts of the locus. Interestingly, only the center of the locus showed clear similarity between fish and mammals, where the genes POLRMT, FBGF 22 or 10 like, BSG, RNF126 and FSTL-3 or similar genes were found (Fig 3). In the various fish species a number of additional serine protease genes that sometimes clustered with chymase locus genes in the phylogenetic tree were seen, and are described later in this communication. In the Nile tilapia and other cichlids, a gene which showed highest homology with granzyme A/K (GzmA-like), was also identified in their met-ase loci (Fig 3).

### The T Cell Tryptase Locus (Also Named the Granzyme A/K Locus)

The T cell tryptase locus appeared to be the best conserved of all the different hematopoietic serine protease loci. It was found in a very similar configuration from cartilaginous fish to humans, with the same flanking genes, although sometimes with more than 2 GzmA/K-related genes (Fig 5). Duplications of the Gzm A gene have occurred in sheep, cattle and rabbit. Additionally in some fish species we observed a massive expansion of this locus with 5 genes in several cichlid species including *Astatotilapia burtoni*, *Haplochromis nyererei* and zebra mbuna (*Maylandia zebra*) with what appears as 13 functional genes and one pseudogene in another cichlid, the Nile tilapia. However, many other fish species have only two copies, as exemplified by the platyfish, zebrafish and spotted gar. In a cartilaginous fish, the elephant shark, five Gzm A/K-related genes were found (Fig 5). However, no genes which showed significant sequence homology to these two serine proteases were identified in the screening of the lamprey, a tunicate and the sea urchin genome, indicating that Gzm A and K, or more correctly their ancestor, appeared at the base of jawed vertebrates.

### Phylogenetic Analyses

**Fig 6:**
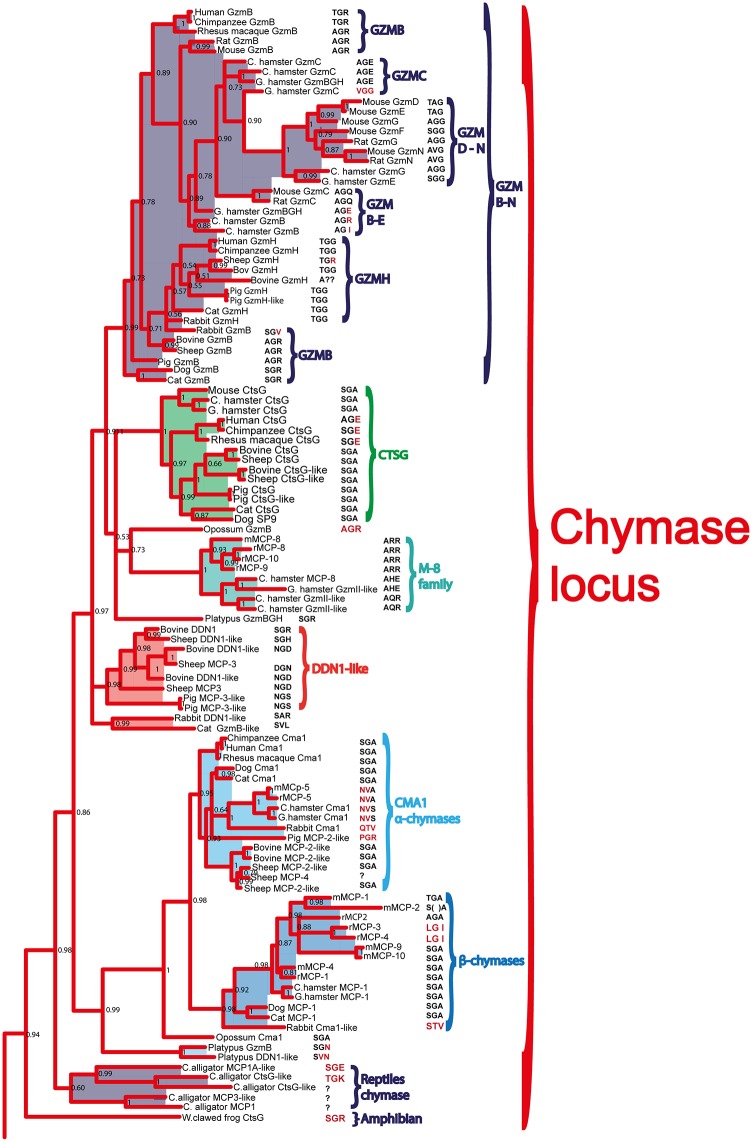
Phylogenetic analysis using the MrBayes algorithm. The original tree includes many members that cannot easily be displayed on one page. The individual parts of the tree have therefore been enlarged and presented separately (Figs 7–10) for the chymase locus-related genes, the fish chymase-related genes, the met-ase locus genes and the granzyme A/K locus genes, respectively. The chymase locus-related fish genes that are found within the met-ase locus (Fig 10) are marked by green instead of blue. Human and mouse coagulation factor X and complement factor B have been used as outgroups to get a more distinct topology of the tree. The three catfish serine proteases that are under more detailed biochemical analyses are marked by red stars in Fig 9. They represent three of the four major sub-branches of fish chymase-locus related serine protease genes. The posterior probability values are shown at each branch point in the tree.

**Fig 7:**
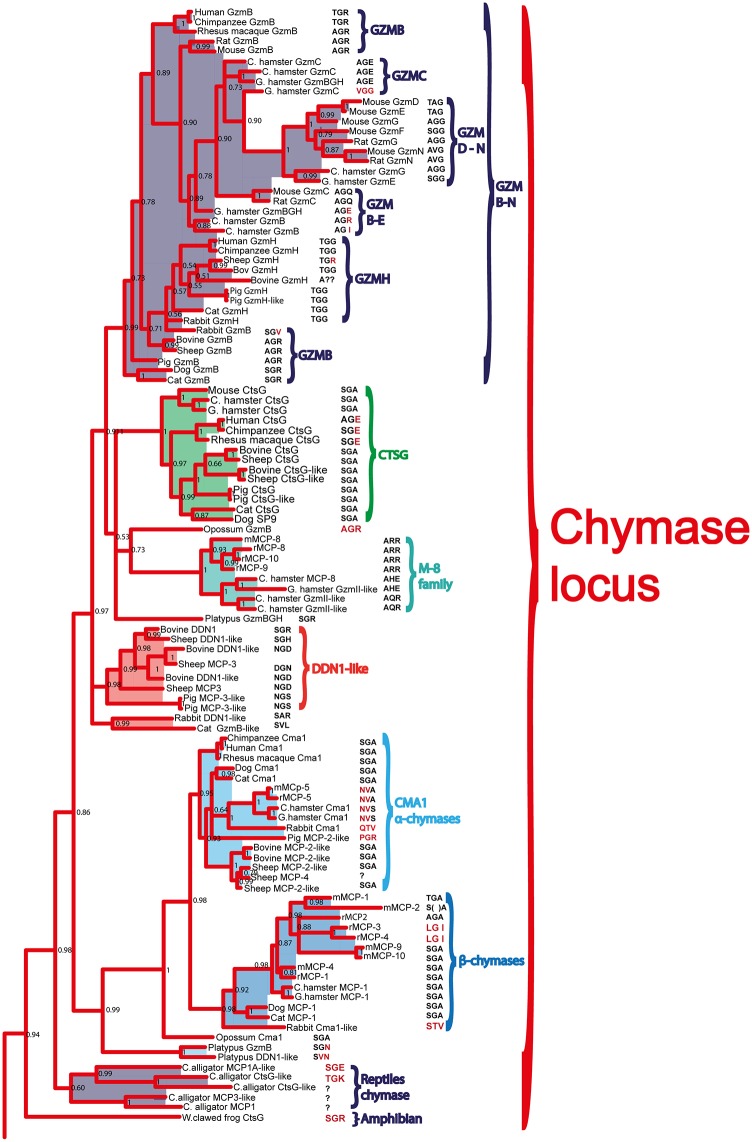
The branch of the phylogenetic tree containing the mammalian chymase locus genes. The triplets constituting the amino acid positions 189, 216 and 226 (S1 pocket), chymotrypsinogen numbering are shown after each of the individual proteases in the tree. A question mark has been inserted when the sequence is not complete, indicating the gene is a pseudogene. The posterior probability values are shown at each branch point in the tree.

**Fig 8:**
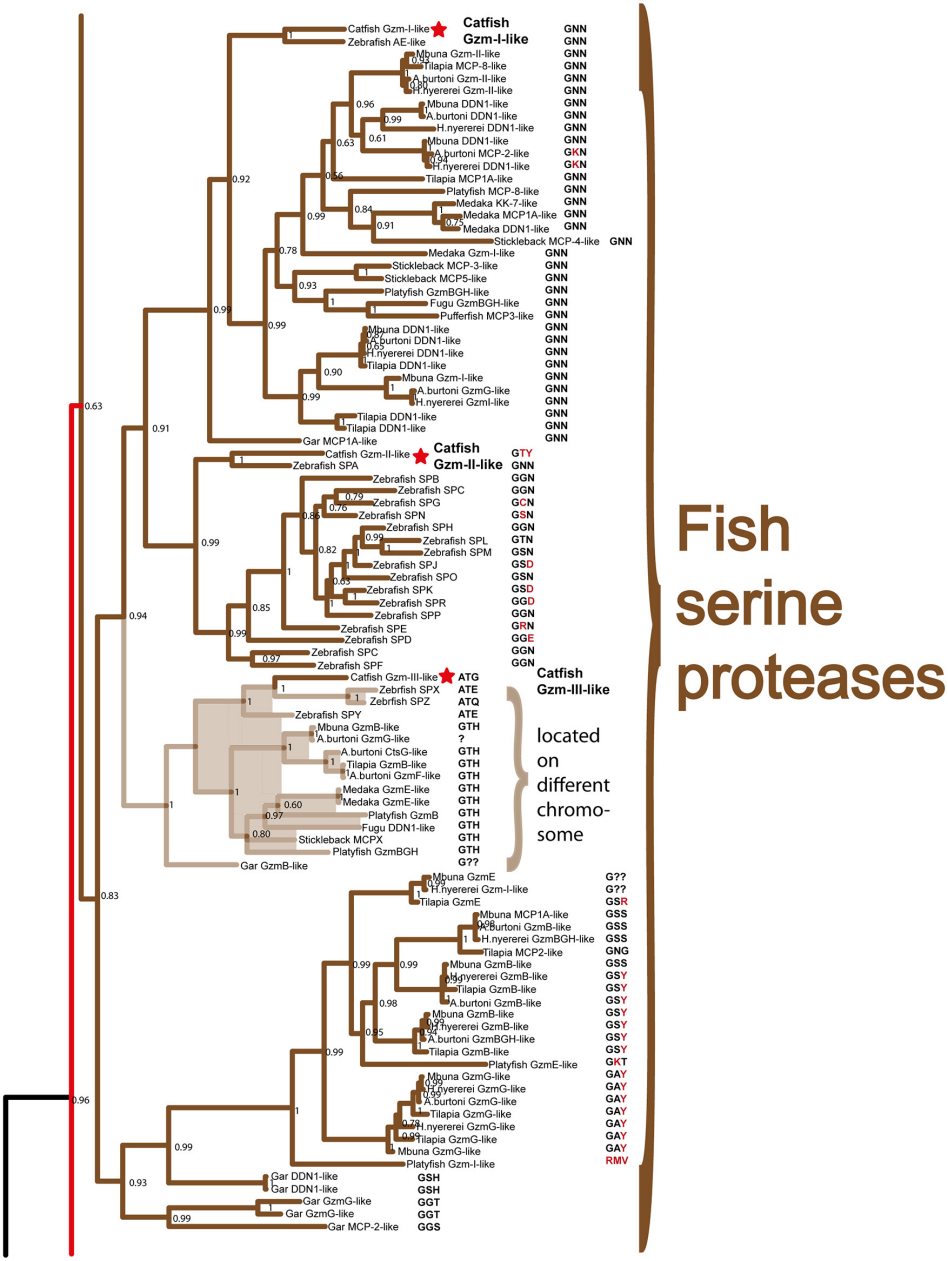
The branch of the phylogenetic tree containing the fish chymase locus related genes. The triplets constituting the amino acid positions 189, 216 and 226 (S1 pocket), chymotrypsinogen numbering are shown after each of the individual proteases in the tree. The posterior probability values are shown at each branch point in the tree.

**Fig 9:**
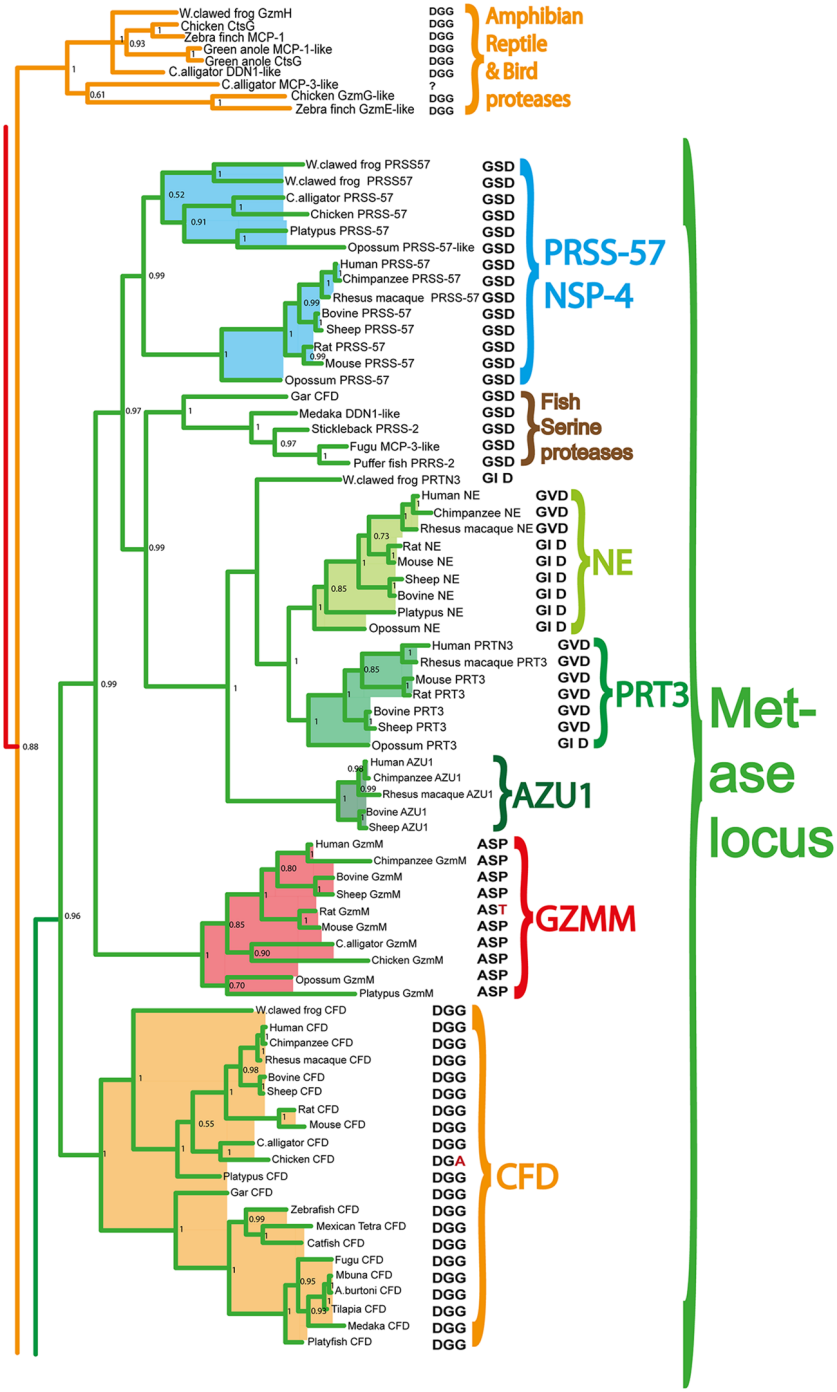
The branch of the phylogenetic tree containing the met-ase locus genes. All the mammalian met-ase locus genes are found within this branch. However, some of the met-ase locus genes in fish are found in the fish chymase locus branch and some in the granzyme A/K branch. The triplets constituting the amino acid positions 189, 216 and 226, chymotrypsinogen numbering are shown after each of the individual proteases in the tree. The posterior probability values are shown at each branch point in the tree.

**Fig 10:**
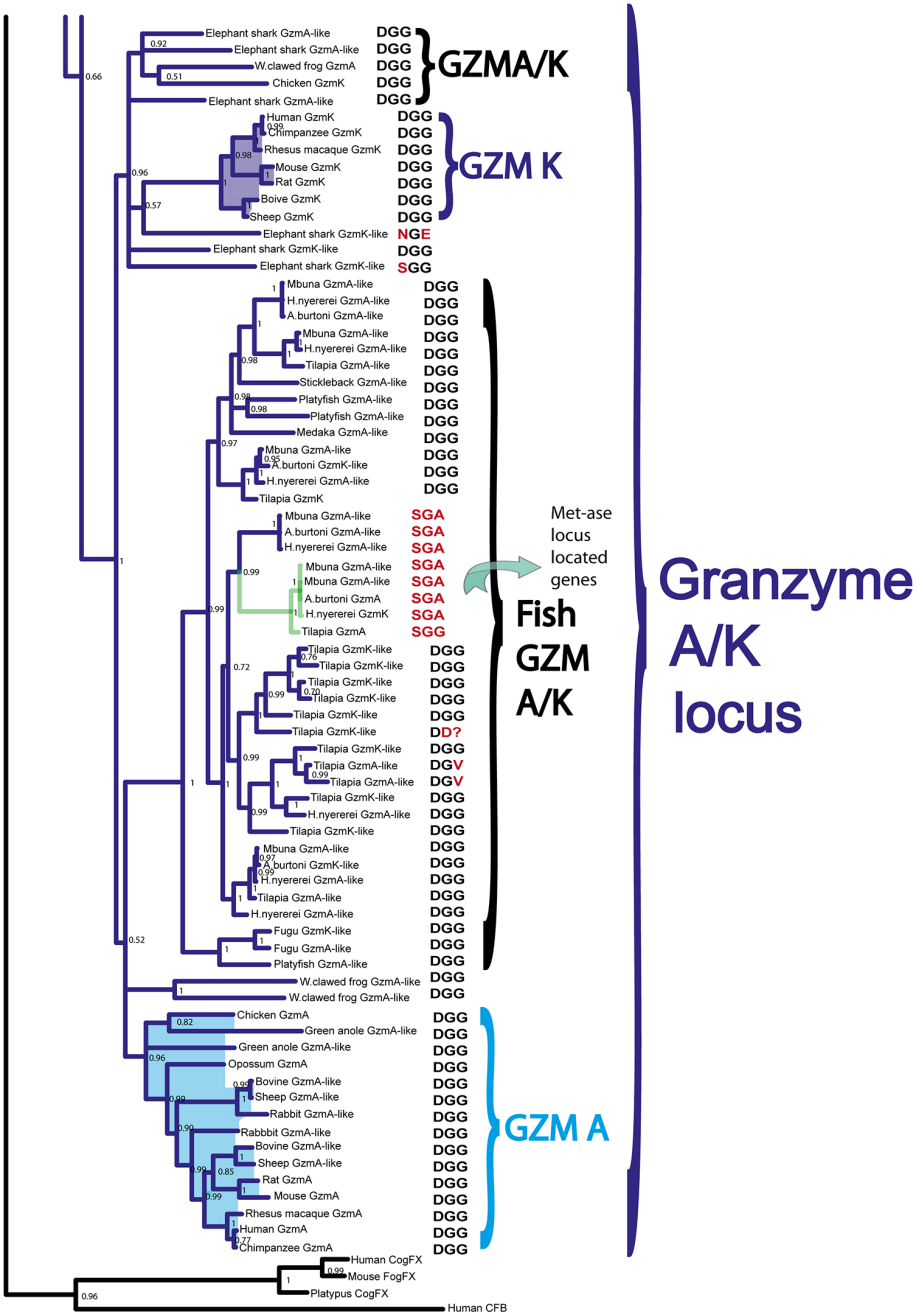
The branch of the phylogenetic tree containing the T cell tryptase locus genes (granzyme A/K locus). The genes originating from the met-ase locus are shown in light blue. The triplets constituting the amino acid positions 189, 216 and 226, chymotrypsinogen numbering, are shown after each of the individual proteases in the tree. The posterior probability values are shown at each branch point in the tree.

The amino acid sequences of the active proteases from the different loci presented in Figs 1–5 were aligned with several different programs to study the relatedness between the various proteases. However, as all the alignments looked very similar only the alignment using MAFFT and the MrBayse program, generating a likelihood phylogenetic tree is depicted (Figs 6–10). The entire tree with associated posterior probability values is shown in Fig 6. Enlargements of the individual branches together with enlargements of the various protease clusters are shown (Figs 7–10) in order to visualize the tree more easily and identifying sub-branches. To verify the data obtained from the MrBayse tree we performed two other alignments using the PHYLIP neighbor joining and PHYLIP maximum likelihood algorithms with associated bootstrap values. These two trees are found as S1 and S2 Figs.

Within the fish branch of genes related to the mammalian chymase locus genes, the ones originating from the potential chymase locus equivalent in fish are shown in black, whereas the genes originating from the met-ase locus are shown in purple (Figs 6 and 8). Interestingly, almost all of the new genes in the fish met-ase loci clustered together with the mammalian chymase locus genes. CFD formed a separate well-defined branch, including members from bony fish to man, indicating a high level of conservation over more than 400 million years (Figs 6 and 9). The branch for PRSS57 contained members from amphibians to man. Two fish proteases clustered in between PRSS57 and the cluster including the separate branches for N-elastase, proteinase 3 and azurocidin (Fig 9). The N-elastase branch included members from platypus to man, proteinase 3 from opossum to man, and the very well defined branch for azurocidin contained only placental mammals (Fig 9). Interestingly, the amphibian (*Xenopus tropicalis*) proteinase 3-like sequence clustered outside the individual branches for N-elastase and proteinase 3, but still close to them, indicating that it originated from a time when these two proteases did not finally separate into individual separate protease genes.

The different Gzm A/K genes formed a separate branch and all the Gzm K genes from placental mammals formed a very distinct subfamily within this tree, outside of all the Gzm A-like genes (Fig 10). Interestingly, the Gzm A/K-related genes within the met-ase locus of the cichlids formed a separate subfamily in the heart of the various Gzm A genes, showing that they were bonafide Gzm A/K genes (Fig 6E). These GzmA/K related genes in the met-ase locus of cichlids are marked by a black small star (Fig 3).

### Analysis of the Residues in the Active Site Pocket Identifying Possible Primary Specificities

**Fig 11:**
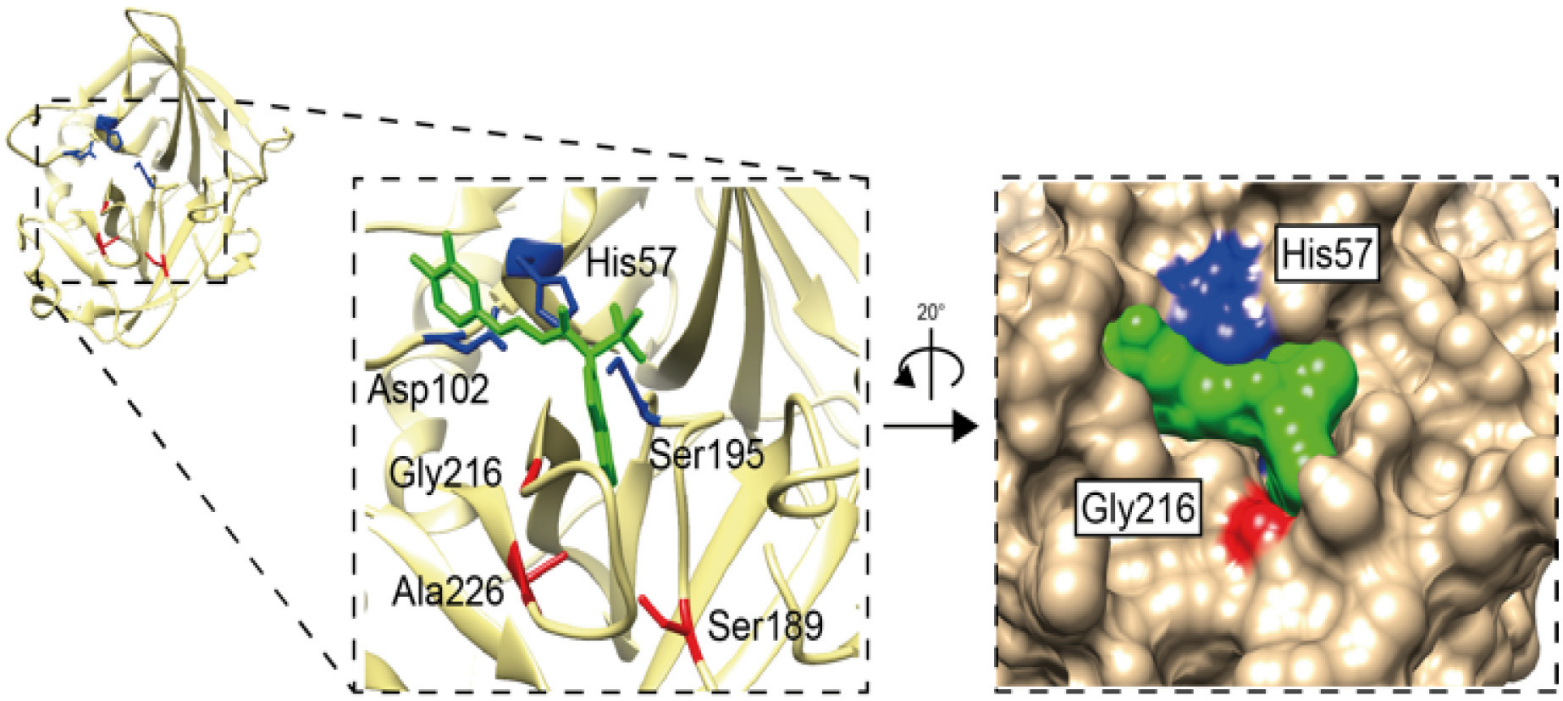
Human chymase structure with inhibitor highlighting the catalytic triad and S1 pocket. The PDB structure 3N7O was used to visualize specificity-conferring triplet (S1 pocket) of residues 189, 216 and 226, chymotrypsinogen numbering, highlighted in red. The catalytic triad residues His57, Asp102 and Ser195 and shown in blue. The bound inhibitor is highlighted in green in both ribbon and space-filling models to visualize how it sits into the S1 pocket. UCSF Chimera program was used to construct the image with further annotation in Adobe Illustrator (CS6).

The pocket of the active site is lined by several amino acids. Three of the most important residues governing the cleavage specificity have been mapped to positions 189, 216 and 226 (comprising the S1 pocket), according to bovine chymotrypsin numbering [26]. This sits adjacent to the so-called catalytic triad, which includes His57, Asp102 and Ser195, providing a mechanism to cleave peptide bonds. Here the Ser provides the name for all serine proteases, which contains such catalytic sites. The catalytic triad and S1 pocket (containing the three specifity-conferring triplet) residues are visualized for the human chymase with bound inhibitor (Fig 11). In tryptases this specificity-triplet often consists of the amino acids Asp, Gly and Gly (DGG), where the Asp in position 189 sits in the bottom of the pocket and provides specificity, hence cleavage, of positively charged amino acids as Arg and Lys. To obtain clues to the primary specificity of the monotreme, fish, amphibian and reptile enzymes we have listed the triplet for each enzyme in the phylogenetic trees (Figs 7, 8, 9 and 10) [26]. A relatively conserved pattern concerning this triplet is seen for the mammalian enzymes. Generally, the tryptases have a triplet of DGG, the chymases SGA (Fig 11), the granzyme Bs that are asp-ases (cleaving after negatively charged amino acids) have SGR, TGR or AGR. Interestingly, the two chymase locus genes in the opossum have SGR and SGA triplets indicating a chymase and a granzyme B homolog. We have previously shown that the opossum chymase is a bona fide chymase with a primary specificity for aromatic amino acids, however, with a slightly different one compared to the homologus mouse (mMCP-4) and human chymases [27]. The opossum enzyme prefers Trp in the P1 position whereas the mouse and human enzymes prefer Tyr and Phe [27]. We have also recently shown that the second opossum enzyme is an asp-ase, although with a more broad extended specificity than the human counterpart (manuscript in preparation). When analyzing the three enzymes in the platypus we find they have triplets of SGN, SVN and SGR (Fig 7). The enzyme with SGR is most likely an asp-ase like granzyme B as it fully matches the sequence of some of the granzyme B’s of placental mammals. The two other genes, which don’t have any exact matches to the consensus triplets of the placental mammalian enzymes, may be a chymase (SGN) and an elastase (SVN). The rodent α-chymases (rMCP-5 and mMCP-5) as well as hamster chymase II that have been shown to be elastases have triplets of NVA and NVS respectively [28–30]. The third platypus enzyme with a SGN triplet, closely matches the SGA of the chymases of placental mammals, which may therefore be a chymase [31, 32]. In reptiles and birds the only species that so far has a chymase locus equivalent is the Chinese alligator (Fig 1). Interestingly the two Chinese alligator sequences that seem fully intact from this locus with no missing exons or stop codons, have triplets of SGK and SGE (Fig 7). The SGK enzyme (CtsG like) is most likely an asp-ase as it most closely matches the SGR of the majority of GzmBs (Fig 7). The second triplet SGE (Alligator MMCP-1A like) is identical to the human and chimpanzee cathepsin G’s, indicating that it may be a chymase, however, with a broader specificity also having a low tryptase activity (Fig 7) [7, 33–35]. One of the other loci found in the Chinese alligator as well as in birds and the green anole lizard encodes genes with a DGG triplet. All the genes within this branch in the phylogenetic tree (Fig 8) have the triplet DGG, which strongly indicates they all are tryptases (Fig 7). The amphibians, as represented by the clawed frog (*Xenopus*), currently has two chymase locus related genes, although the genome is still incomplete. One of them clusters with the mammalian chymase locus and the other with the reptile and bird locus described above that encodes tryptase like genes. This second gene, which is named GzmH in the clawed frog, has the same triplet as the other genes in this branch of the tree, DGG, and is thereby most likely a tryptase. Interestingly, the other gene that is more closely related to the mammalian enzymes has a triplet identical to some of the mammalian granzyme B’s, namely SGR, indicating that it is an early granzyme B homolog. This indicates that granzyme B was the first enzyme to appear in this locus. The specificity of the different chymase-related fish proteases are more difficult to assign on the basis of these triplets. They are somewhat different to the ones described and also have a large number of varying triplets but a few are dominating including GNN, GGN, GTH, GSS, GAY and GSY (Fig 8).

The met-ase locus genes are more easily analyzed as the triplet patterns are highly conserved. All the CFDs have triplets of DGG except in the chicken, which has DGA, indicating that all of them are tryptases, as their mammalian members [36]. All the met-ases, including ones from alligators, chickens and mammals have a triplet of ASP, except rat GzmM that has AST, indicating that they all are met-ases (Fig 9). All the Prss57 (NSP-4) genes, including the related fish proteases, have a triplet of GSD [8]. The human variant has been shown to be a tryptase suggesting that all the other enzymes also are tryptases. Interestingly, all the N-elastase and proteinase 3 sequences including the *Xenopus* sequence have triplets of GVD or GID (Fig 9). N-elastase and proteinase 3 primarily accommodate small hydrophobic residues in the S1 pocket of the enzyme and thereby determined to be elastases [37]. This also highlights that caution should be taken for genes originating from this locus while drawing conclusions only based on this triplet for these enzymes. The azurocidins are catalytically inactive proteases, therefore no analysis of these triplets are of any significance.

In the GzmA/K locus almost all enzymes, from sharks to humans, have DGG triplets. However, all the five cichlid genes that are found in the met-ase locus, which cluster in the middle of the GzmA/K-related genes (Fig 10) have a different triplet, SGA, a typical chymase triplet, indicating they have changed specificity and have now become chymases. Three additional cichlid genes that cluster closely to these genes also have a SGA triplet. These are located in the middle of the classical A/K locus in these cichlid species (marked by red stars in Fig 5). Two of the five GzmA/K-related genes in the elephant shark also have triplets that do not conform to the general DGG, one with a NGE and one with SGG (Fig 10). The SGG enzyme may also be a chymase whereas the NGE enzyme is more difficult to assign a primary specificity based on only this triplet.

## Discussion

Through a detailed analysis of the genes related to three of the four loci encoding the various hematopoietic serine proteases in mice and humans, we have been able to show that the first clearly identifiable members of this big class of proteases appeared with the cartilaginous fish. However, the mast cell tryptase locus may also be an early locus but outside the scope of this analysis. Analyzing this locus involves other difficulties, as many trypsin-related gene clusters are present in most organisms and it is often difficult to clearly identify their origin, thereby labeling them as the true tryptase locus of lower vertebrates or chordates.

In our analyses, the Gzm A/K locus was not only the first to appear but also the most conserved out of three analyzed. In the elephant shark, a Gzm A/K locus was found that essentially looks like its mammalian counterpart with only one major difference being the number of GzmA/K like genes. In humans there is one gene for Gzm A and one for Gzm K whereas in the elephant shark there are 5 different genes that are closely related to mammalian A and K. Interestingly, the granzyme Ks of placental mammals formed a separate subfamily outside the entire A branch (Fig 10). At the protein level, granzyme K forms monomers due to the lack of an extra cysteine in position 93 (chymotrypsinogen numbering) of the active human granyzme K, whereas granzyme A forms dimers due to the presence of this unpaired cysteine [38, 39]. When we look at all other animal classes, chickens and green anole lizards followed this mammalian pattern with a granzyme K that lacked a cysteine and a granzyme A that had one. In the green anole, the two genes were both named Gzm A-like although one was more K-like due to the lack of this cysteine. The same pattern was seen in frogs (Western clawed frog) where two out of three genes lacked this cysteine, and also in elephant sharks where four out of five genes lacked the cysteine. Bony fish had both variants, indicating that the two types are old, although the functional consequences of having two variants are still not known. One possible explanation could be related to the accessibility for substrates as a dimer most likely has a more restricted active site in comparison to a monomer. Interestingly, there is also a major controversy concerning the functions of these two proteases. For a long time they were thought to be primarily important for apoptosis induction in virus infected cells and other cells targeted for cell death by cytotoxic T cells and NK cells [40]. However recently, this model has been questioned as using more physiological concentrations of the proteases and testing for a biological response results in no or only minor apoptosis induction but rather a potent cytokine response [41]. These studies therefore indicate that the induction of cytokine expression and inflammation may be the major role of these proteases. One additional very interesting finding was the presence of a bonafide Gzm A/K gene in the met-ase locus of all cichlid species analyzed but not in other bony fishes (Fig 3). This gene seems to have been inserted, by a yet unknown mechanism, into the middle of a cluster of genes distantly related to the chymase locus genes in mammals that sit in the met-ase locus of fishes (Fig 3).

The met-ase locus was the second most conserved locus of the four hematopoietic serine protease loci identified in mammals. Here, the same bordering genes from fish to mammals were found, including the same non-protease genes in the center of the region (Fig 3). However, and in contrast to the Gzm A/K locus, the number of genes and types of genes in the met-ase locus differed significantly between bony fish and mammals (Fig 3). The only gene that seemed to be present in all jawed vertebrate lineages except cartilaginous fish was CFD. However this was also not located in the same place in all bony fish species and lineages. This gene was present in the spotted gar met-ase locus but not in the zebrafish, medaka, stickelback, platyfish or the different cichlids (Figs 3 and 4). Interestingly, the CFD gene in these latter species was found in another locus (as seen in Fig 4). It is difficult to say in which of these two loci was the original position for CFD. In the spotted gar and platyfish another gene that clustered with the met-ase locus genes in mammals was also found, named MCP3 and CFD-like, respectively, which formed a branch between PRSS57 and the three other neutrophil expressed genes; proteinase 3, N-elastase and azurocidin (Fig 9). This is the first clearly identifiable member of the different neutrophil proteases, which indicates that they appeared with bony fish. In amphibians (Western clawed frog) there were additional genes which more clearly corresponded to the various mammalian met-ase locus genes, including a PRSS57 gene and also an early variant of the gene that most likely later duplicated and formed proteinase 3 and N- elastase, and probably also azurocidin in mammals (Fig 3). However, no gene for GzmM was found in amphibians, which was first observed in reptiles and birds, as exemplified by the Chinese alligator and chicken. A likely scenario is that this gene appeared with early reptiles. In the reptile lineage there has been a loss of genes corresponding to N-elastase and proteinase 3. No gene with clear homology to these two genes is found in the chicken or Chinese alligator. However, these two genes are clearly identifiable in the opossum, a marsupial. The platypus genome is still incomplete and it’s possible they are also present there. In a very recent update on the platypus genome sequence Gzm M, PRSS57, CFD and N-elastase were found on different contigs, although no gene for proteinase 3 was identified (Fig 3).

The chymase locus is the most complicated of the three we have analyzed in this communication. A clearly defined chymase locus is so far only seen in mammals. In all placental mammals and in the marsupials, as exemplified by the opossum, we have a chymase locus with the same or at least partly the same bordering genes. In some species, one side of the locus has suffered translocations, as exemplified by the mouse, which has one end with other bordering genes and the ruminants, cow and sheep that have the other end of the locus with other bordering genes (Fig 1). In the opossum an inversion has occurred which has moved the two genes of the locus, the chymase and a Gzm B homolog, apart. This inversion may have hampered further expansion of the locus due to a lack of sequence homology that can facilitate unequal crossing over events. In the still incomplete platypus genome, three closely-related genes are also found on two short contigs suggesting a similar locus is probably also present in monotremes. Interestingly, two of these three genes cluster with the chymase subfamily and one of them with the branch containing granzymes and cathepsin G (Figs 1, 6 and 7). This tells us that these two subfamilies within the chymase locus were already present during early mammalian evolution. When we come to reptiles and birds we find genes that cluster close to the mammalian chymase locus genes in the phylogenetic tree but have completely different bordering genes (Fig 1). However, in one of the species, the Chinese alligator, we find four contigs that cluster with the chymase locus genes and three of them showed closer homology to the mammalian chymase locus genes than the fourth contig (Figs 1, 6, 7 and 8). No bordering genes were present on these three contigs, which makes it difficult to tell its relation to the mammalian chymase locus. However, it gives good indications that a chymase locus was present in early reptiles, which has possibly been lost in most reptile and bird lineages and instead been replaced by genes within another locus that have possibly taken over roles performed by the genes of the mammalian chymase locus genes by convergent evolution. In the *Xenopus tropicalis*, we find two genes related to the mammalian chymase locus. However, currently there are no bordering genes that can be used to identify their relatedness to the alligator or mammalian loci. One of the genes does, however, cluster closely to the mammalian genes, indicating that it is an early version of the mammalian locus. When analyzing fishes the pattern is even more complicated. There we have another set of chymase locus-related genes that has different bordering genes from both the mammalian chymase locus and the locus we find in reptiles and birds. In addition, in some fish species we also have a large number of chymase locus-related genes in the met-ase locus (Fig 3). In the most recent update of the zebrafish genome there are as many as 19 serine protease genes in the met-ase locus and the majority of these cluster in a large fish specific branch close to the chymase locus genes of mammals (Figs 3, 6 and 8). The question then arises of the evolutionary origin of these genes. The met-ase locus in some fish does not only harbor chymase locus-related genes but also, as mentioned above, a bonafide Gzm A/K gene as observed in all the cichlids analyzed (Figs 3, 6 and 10). However, these GzmA/K genes may have changed primary specificity and become chymases as they have the triplet SGA (Fig 10). The chymase locus-related genes may have developed by convergent evolution but the origin of these Gzm A/K genes in the cichlids is more complicated. How has a gene that is so closely related to Gzm A/K of mammals come to be positioned in the met-ase locus and there within a cluster of chymase locus-related genes and there changed primary specificity? The large evolutionary distances make a detailed analysis of mechanisms very difficult as it becomes almost impossible to trace sequence elements that may have been involved in potential recombination events when spanning over large evolutionary distances so it may remain an unanswered question.

Summarizing the findings concerning the evolution of these three loci appears as they are only found in jawed vertebrates and that only one of them, the granzyme A/K locus can be traced as far back as in cartilaginous fish. The met-ase locus is then clearly present in bony fish, with the same bordering genes as in mammals and also containing CFD, at least in some fish species, and also a gene related to the neutrophil proteases in this locus. However, the situation concerning the chymase locus is more complex and may involve genes from several loci and convergent evolutionary processes.

Gene duplications have been a major player in the evolution of these proteases subfamilies and loci, and unequal crossing over has most likely been the domination process. However, other mechanisms are probably also involved. The explanation for the apparent movement of the grzA/K gene from the classical GzmA/K locus to the met-ase locus is an example of the latter example, where we still have few good explanations for the mechansim. A very interesting example of gene evolution involving this protease family is the appearance of a new class of antifreeze proteins in antarctic fishes. There a gene for a trypsin related serine protease has been the frame for the generation of a highly repetitive antifreeze protein where a tree amino acid repeat now make up almost 100% of the protein and the only remaining part of the original trypsinogen gene is the signal sequence and the 3´non coding exon [42]. The entire central part of the gene now consists of this tree amino acid repeat.

We recently published an article presenting a detailed analysis of the appearance of Fc receptors for immunoglobulins during vertebrate evolution and there observed a major step in the evolution of adaptive immunity at the base of bony fish [43]. It is well established that immunoglobulins (Igs) and T cell receptors (TCRs) appear at the base of jawed vertebrates, as no Igs and TCRs are found in the hagfish and lamprey [44]. These two fish species represents two lineages of jawless fishes. However, what we found was that no genes similar to any of the classical Fc receptors, the Fc receptor-like molecules (FcRL) including the transport receptor for IgA (PIGR) or the signaling component FcR gamma chain or any of the NK cell receptors including KIRS and LILRs were found in cartilaginous fish, as they seem to first appear with the bony fish [43]. When we now look at the hematopoietic serine proteases we also see major developments occurring at the base of bony fish. So far it is only the GzmA/K locus that is present in cartilaginous fish. With these two studies it seems as cartilaginous fish, as also seen with the jawless fish, have solved several important questions related to immunity in a very different way than other vertebrates. How do the Igs of cartilaginous fish interact with immune cells; how are NK cells or NK-like cells activated and how do their immune cells solve the problem of apoptosis induction in target cells? For example, do they have other proteases that serve the function of granzyme B in apoptosis? Almost nothing is yet known about these important issues in cartilaginous fish. More is known about these proteases in bony fish but also here major questions remain. The chymase locus-related genes in fish form a separate branch in the phylogenetic tree and this branch has at least five major sub-branches. The only species where a more detailed analysis has been performed concerning these proteases is in the channel catfish. There, four proteases have been identified and they have been cloned from different cell lines. These enzymes named catfish SP1, 2 and 3 and Gzm A are being expressed in catfish NK, cytotoxic T lymphocytes and macrophage cell lines [45, 46]. One major issue in this field is now to study the functions, the tissue distributions and cleavage specificities of these proteases, and if they have developed similar functions as their mammalian counterparts or solved the problems of immune protection by other mechanisms?

A first step in identifying the primary cleavage specificity of a protease and thereby the potential function is by looking at the residues of the pocket binding the P1 position of the substrate. The residues 189, 216 and 226 (chymotrypsinogen numbering) constitute the S1 pocket, which can be used to gain clues to the primary specificities of these enzymes. The absolute majority of the tryptases have a triplet consisting of the three amino acids DGG, where the Asp in the 189 position, which is located in the bottom of the pocket, favors positively charged amino acids in the P1 position of substrates [26]. The absolute majority of the granzyme A/Ks are tryptases and almost all have a triplet of DGG (Fig 10). There are a few exceptions where the Asp in position 189 has been changed to a Ser and the Gly in position 226 to an Ala resulting in the triplet SGA. All five Gzm A/K-related genes in the met-ase locus, except one that has SGG, have this sequence indicating they have changed specificity to become chymases. Interestingly, three additional genes from the same cichlid species have changed the 189 position to a Ser and these are the most closely related to the five genes of the met-ase locus according to the phylogenetic tree (Fig 10). This indicates that the copies of these genes have moved to the met-ase locus by some yet unknown mechanism.

The majority of the chymases have a triplet of SGA, the same triplet as the non-canonical GzmA/K genes described above. The small non-charged amino acids in this triplet favor large hydrophobic amino acids like the aromatic amino acids Tyr, Phe, and Trp, as well as Leu. However, the selectivity can change quite dramatically from one chymase to another. rMCP-2, the major rat mucosal mast cell protease is a very active protease that prefers Tyr and Phe in the P1 position and does not tolerate Trp, whereas the opossum chymase favors Trp over the other two aromatic amino acids [27, 47].

The asp-ases (Granzyme B) often have a triplet of AGR or TGR where the Arg in the 226 position results in a preference for negatively charged amino acids in the P1 position. Mutation of this residue into an Asp changes the primary specificity of the enzyme into a tryptase with a primary specificity for Arg or Lys [48]. Interestingly the *Xenopus* CTSG and the two alligator sequences, CTSG-like and mMCP-1A like that are most closely related to the mammalian chymase locus genes have triplets of SGR, SGK and SGE, respectively. These three proteases may represent early ancestors of the chymase locus in mammals. The SGR and the SGK triplets of the *Xenopus* CTSG and alligator CTSG indicate they have similar primary cleavage specificities to human and mouse granzyme B and therefore are asp-ases, whereas the second alligator sequence, mMCP-1 like, with a triplet of SGE, may have a specificity similar to human cathepsin G. Human cathepsin G, which has the triplet AGE, has been shown to have a broader specificity, being primarily a chymase but with an additional lower tryptase activity [7, 33–35]. Here, the Glu in position 226 may be of importance for this activity. However, the position 226 does not seem to be as important for predicting the primary specificity as the position 189, as residue 226 can sometimes be shielded by neighboring residues [26]. When looking at the triplets of the various fish proteases it is only the granzymes A and K that are easy to predict as they generally have identical or very similar triplets as their mammalian counterparts (DGG) (Fig 8). However, for the other fish proteases the picture is much less clear, which is most likely due to the larger evolutionary distance hence are more structurally different from the mammalian enzymes. The first group within the fish protease group (Fig 8) which includes catfish I, zebrafish AE-like and Gar MCP-1A like, generally have a triplet of GNN. This triplet has no direct homolog among the mammalian enzymes and is therefore difficult to predict a primary specificity based on this triplet. We have recently performed a phage display analysis of the catfish I enzyme belonging to this group of proteases and seen that it’s the most specific hematopoietic protease identified so far. The protease is a met-ase with a very well defined extended specificity and a consensus cleavage sequence of (RVTGMSLV) (manuscript in preparation). This sequence was not possible to have been predicted based only on the GNN triplet of this enzyme. We have also performed phage display on one additional fish protease, the catfish II enzyme, belonging to the second subfamily within the fish proteases (presented in Fig 8) (manuscript in preparation). This enzyme has a triplet of GTY indicating that it may be an elastase due to the large hydrophobic amino acid in the position 226. However, what we observed is a highly specific tryptase with specificity for multiple positively charged amino acids; RR, RRR or RRRR (manuscript in preparation). The catfish III also seems to be highly specific as we did not see any cleavage of a battery of chromogenic substrates representing tryptase, chymase, elastase and asp-ase substrates. A similar situation was observed for catfish I and II when running the chromogenic substrates. Fish proteases appear to have a more defined extended specificity compared to most of the mammalian enzymes and it is much more difficult to obtain an accurate estimate of their primary specificity based only on these three amino acids. In light of this high specificity for the fish proteases it is interesting to note that they have the extra cysteine bridge Cys191-Cys220, which is lacking in the mammalian proteases originating from the chymase locus. This may partly explain the more restricted specificities of these proteases where larger, open and flexible binding sites for substrates in the mammalian enzymes are observed [49].

In order to obtain a more detailed view of the function and diversification of the various novel fish proteases identified in this screening we plan to look at the primary and extended specificities of several additional members of these enzymes in future studies. We also plan to study some of the tetrapod sequences from the platypus, alligator, chicken and the *Xenopus* to determine their primary and extended specificities. Here, it will be interesting to see if the predictions based on similarities in these triplets also prove to be correct when tested experimentally.

During the completion of this study a similar study was published confirming many of the conclusions concerning the genomic organization of these genes [50]. The identification of a GzmA/K locus in cartilaginous fish, an ancestral met-ase locus in bony fish, the three platypus chymase locus genes and also the genes related to the chymase locus in frogs was confirmed in this article. The combined information from these two studies can now form a solid framework for a more detailed analysis of the evolutionary origin of these proteases, as well as their primary and extended cleavage specificities, and how they contribute to immunity in species from different parts of the vertebrate evolutionary tree.

## Supporting Information

S1 Fig Phylogenetic Tree constructed using the PHYLIP program and the neighbor joining algorithm. Bootstrap values after 100 replicates are included at all branch points.
(TIF)

S2 Fig Phylogenetic Tree constructed using the PHYLIP program and the maximum likelihood algorithm. Bootstrap values after 100 replicates are included at all branch points.
(TIF)

S1 File Protein Accession numbers of vertebrate proteases (DOCX)

## Abbreviations

mMCP: mouse mast cell protease
rMCP: rat mast cell protease
NSP-4: neutrophil serine protease 4
CFD: complement factor D
