# Correction: Granule Associated Serine Proteases of Hematopoietic Cells – An Analysis of Their Appearance and Diversification during Vertebrate Evolution

**DOI:** 10.1371/journal.pone.0145592

**Published:** 2015-12-22

**Authors:** Srinivas Akula, Michael Thorpe, Vamsi Boinapally, Lars Hellman

The image for Fig 7 was incorrectly duplicated as Fig 6. Please view the correct Fig 6 here.

**Fig 6:**
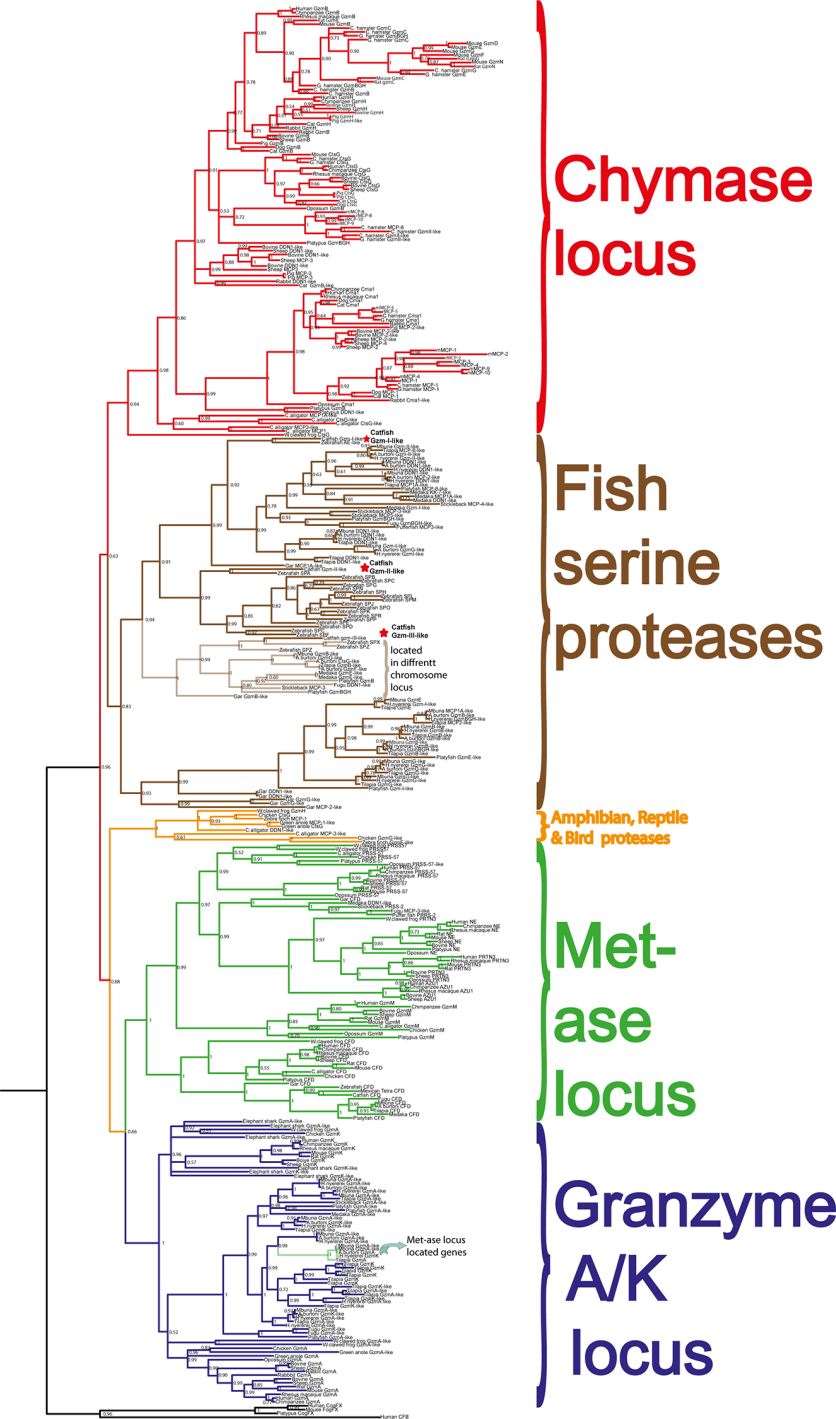
Phylogenetic analysis using the MrBayes algorithm. The original tree includes many members that cannot easily be displayed on one page. The individual parts of the tree have therefore been enlarged and presented separately (Figs 7–10) for the chymase locus-related genes, the fish chymase-related genes, the met-ase locus genes and the granzyme A/K locus genes, respectively. The chymase locus-related fish genes that are found within the met-ase locus (Fig 10) are marked by green instead of blue. Human and mouse coagulation factor X and complement factor B have been used as outgroups to get a more distinct topology of the tree. The three catfish serine proteases that are under more detailed biochemical analyses are marked by red stars in Fig 9. They represent three of the four major sub-branches of fish chymase-locus related serine protease genes. The posterior probability values are shown at each branch point in the tree.
