# Supplementary figures and images for: Granule Associated Serine Proteases of Hematopoietic Cells – An Analysis of Their Appearance and Diversification during Vertebrate Evolution
Source: PLoS One. 2015 Nov 16;10(11):e0143091. doi: 10.1371/journal.pone.0143091 (PMC4646688; doi:10.1371/journal.pone.0143091)

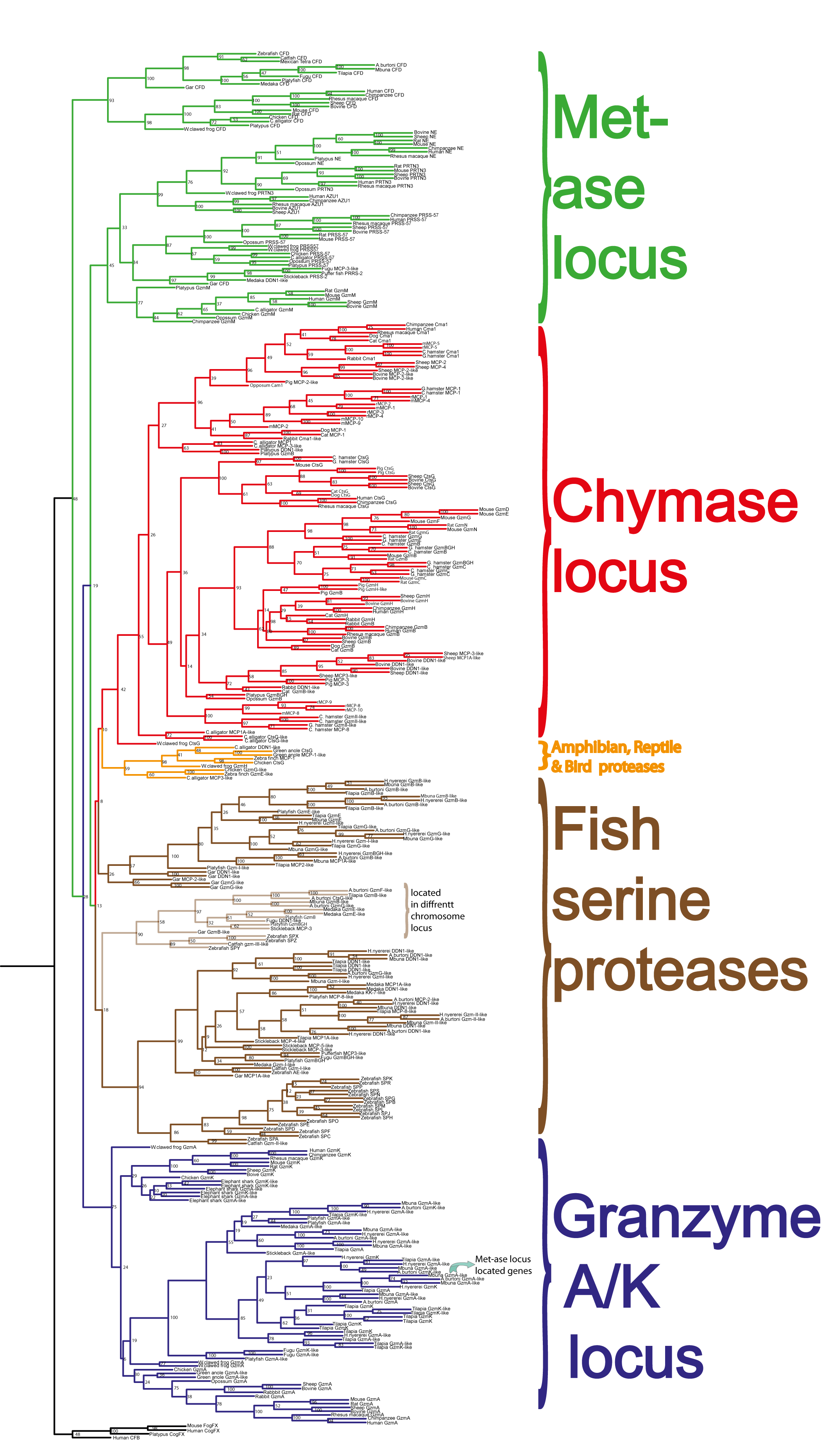

Supplement: S1 Fig — Bootstrap values after 100 replicates are included at all branch points. (TIF) [file pone.0143091.s001.tif]

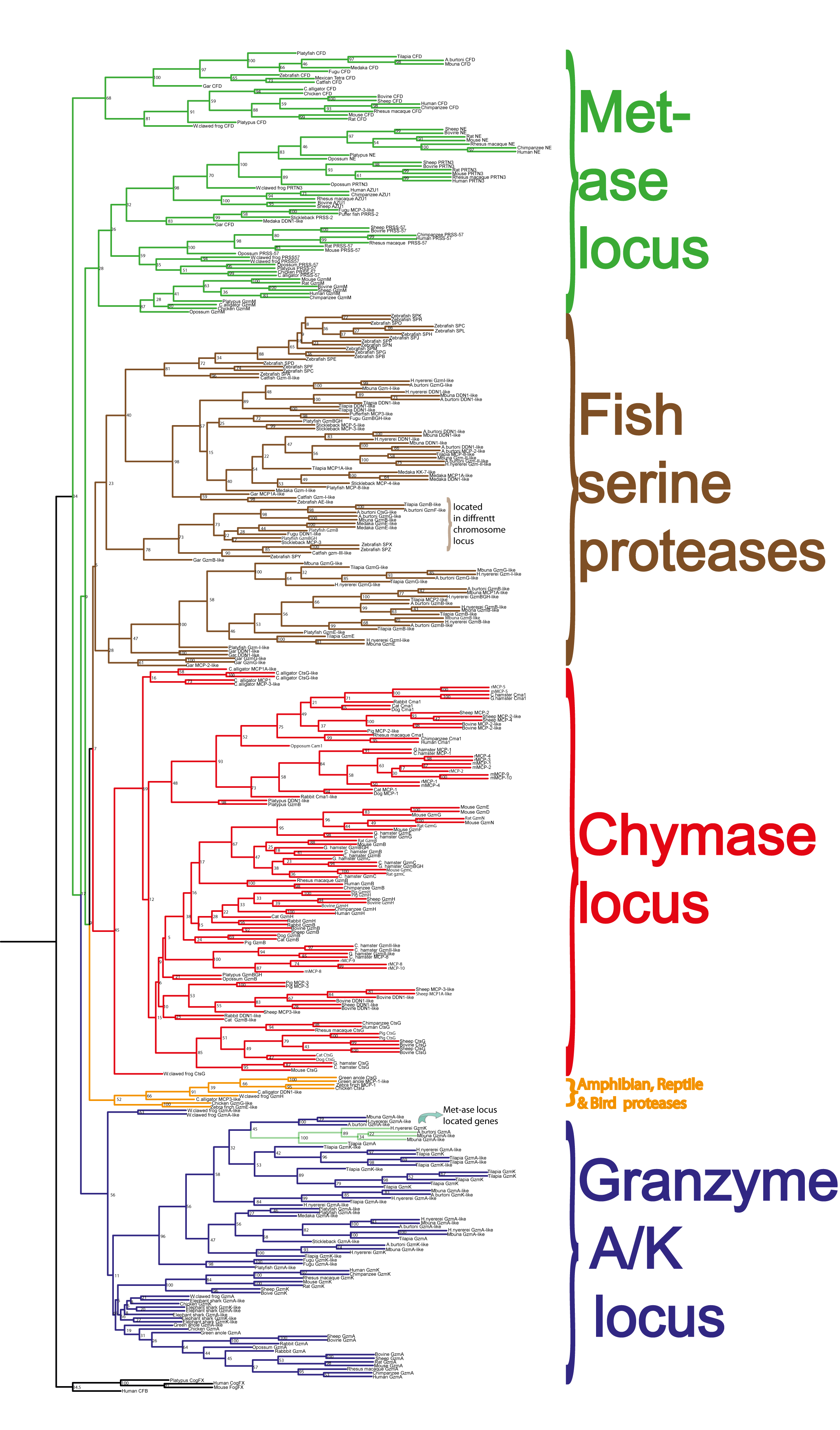

Supplement: S2 Fig — Bootstrap values after 100 replicates are included at all branch points. (TIF) [file pone.0143091.s002.tif]
