# Supplementary material for: Granule Associated Serine Proteases of Hematopoietic Cells – An Analysis of Their Appearance and Diversification during Vertebrate Evolution
Source: PLoS One. 2015 Nov 16;10(11):e0143091. doi: 10.1371/journal.pone.0143091 (PMC4646688; doi:10.1371/journal.pone.0143091)
Supplement: S1 File — (DOCX) [file pone.0143091.s003.docx]

S1 file

Protein Accession numbers of vertebrate proteases

Human: GzmB (NP_004122), GzmH (NP_219491), CMA1 (NP_001827), CtsG (NP_001902), GzmA (NP_006135), GzmK (NP_002095), GzmM (NP_005308), CFD (NP_001919), PRSS57 (NP_999875), NE (NP_001963), PRTN3 (NP_002768), AZU1 (NP_001691), Factor X (AAA52486) and Complement Factor B (AAA16820).

Chimpanzee: GzmB (XP_509879), GzmH (XP_522811), CMA1 (XP_001170224), GzmA (XP_001148261), GzmK (XP_527196), CFD (XP_003953351), PRSS57 (XP_001146596), NE (XP_009432529) and AZU1 (XP_524023).

Rhesus macaque: GzmB (XP_001114420), CMA1 (NP_001181562), GzmA (XP_001097639), GzmK (NP_001253239), CFD (XP_005587397), PRSS57orSP1 (EHH29398), NE (XP_005587396), PRTN3 (XP_001117176) and AZU1 (XP_005587393).

Rat: GzmB (granzyme-like-protein-1) (NP_612526), GzmC (NP_599159), GzmH (G) (NP_703196), GzmN (NP_001178045), rMCP-1 (NP_001264597), rMCP-2 (NP_742041), rMCP-3 (NP_001163937), rMCP-4 (NP_062194), rMCP-5 (CMA1) (NP_037224), rMCP-8 (NP_067609), rMCP-9 (NP_062196), rMCP-10 (grazyme-likeII) (NP_058842), GzmA (NP_703198), GzmK (NP_058815), GzmM (NP_476531), CFD (NP_001071110), PRSS57 (XP_006241041), NE (NP_001100237) and PRTN3 myeloblastin (NP_001019435).

Mouse: GzmB (NP_038570), GzmC (NP_034501), GzmD (NP_034502), GzmE (NP_034503), GzmF (NP_034504), GzmG (NP_034505), GzmN (NP_694692),

CtsG (NP_031826), mMCP-1 (NP_032596), mMCP-2 (NP_032597), mMCP-4 (NP_034909), mMCP-5 (CMA1) (NP_034910), mMCP-8 (NP_032598), mMCP-9 (NP_034912), GzmA (NP_034500), GzmK (NP_032222), GzmM (NP_032530), CFD (NP_038487), PRSS57 (NP_001036175), NE (NP_056594), PRTN3 (NP_035308) and Factor X (NP_001229297).

Rabbit: GzmA-like (XP_002714023), GzmA-like (XP_002714079), CMA1 (XP_002717970), CMA1-like (XP_002717971), GzmH (XP_008267676), GzmB (XP_002718156) and DDN1-like (XP_008267677).

Bovine: MCP1A (XP_002696733), CtsG (XP_581980), GzmA (NP_001092565), GzmA (NP_001001142), GzmK (NP_001192888), MCP-2 (XP_593156), MCP-2 (XP_001790127), CtsG (XP_587026), GzmH (XP_874639), DDN1-like (XP_001252849), DDN1 (XP_585694), GzmB (XP_585453), GzmM (XP_589755), CFD (NP_001029427), PRSS57 (NP_001289871), NE (NP_001099123), PRTN3 (XP_002689189) and AZU1 (XP_002689188)

Sheep: GzmB-like (XP_004022950), GzmH-like (XP_004018154), CtsG-like (XP_004018151), CtsG-like (XP_004018152), DDN1-like (XP_004018153), sMCP-3-like (NP_001009411), sMCP-3-like (XP_004017918), sMCP1A-like (NP_001009472), sMCP-2 (NP_001116477), sMCP-4 (NP_001009757), GzmA-like (XP_004017040), GzmA-like (XP_004017039), GzmK (XP_004017041), GzmM (XP_004009511), CFD (XP_004008816), PRSS57 (XP_004009516), NE (XP_004009521), PRTN3 (XP_004009520), and AZU1 (XP_004009519).

Pig: MCP-2-like (XP_005666287), MCP-3-like (XP_003482301), CtsG-like (XP_001926799), GzmH (NP_001137165), GzmB (NP_001137182), CtsG-like (XP_005666288), GzmH-like (XP_005666278) and MCP-3-like (XP_005666276) and AZU1 (XP_005661477).

Dog: CMA1 (NP_001013442), MCP-1 (XP_003435162), TMPRSS9 or CtsG-like (XP_005623965) and GzmB-like (XP_547752).

Cat: CMA1 (XP_003987574), MCP-1-like (XP_003987617), CtsG (XP_003987618), GzmH (XP_006932855), GzmB-like (XP_006932853) and GzmB (XP_006932885).

Chinese Hamster: CMA1-like (XP_007640852), MCP-1-like (XP_003502094), MCP-8-like (XP_003502093), GzmII-like (XP_003502092), GzmII-like (XP_003502091), CtsG (XP_003502127), GzmG-like (XP_003502126), GzmC-like (XP_003502090), GzmB-like (XP_003502089), GzmC-like (XP_003502088) and GzmB (XP_003502125).

Golden hamster: CMA1 (XP_005085683), MCP-1-like (XP_005085684), GzmII-like (XP_005085699), CtsG-like (XP_005075410), GzmE-like (XP_005075409), GzmC-like (XP_005075408), GzmBGH-like (XP_005075407) and GzmBGH-like (XP_005075406).

Opossum: CMA1 (XP_001369716), GzmB (XP_001369757), GzmA (XP_007486373), GzmM (XP_001375908), PRSS57 (XP_007489505), PRSS57-like (XP_007489487), NE (XP_001379966) and PRTN3 (XP_001379973).

Platypus: GzmB (NP_001229635), DDN1-like (XP_001512980), GzmBGH (XP_007660654), CFD (XP_003429544), PRSSP57 (XP_001519084), NE (NP_001121095) and Coagulation factor X (NP_001121086)

Chicken: CtsG (DDN-1) (XP_423728), GzmG-like (XP_004948782), GzmA (NP_989788), GzmK (XP_423832), GzmM (XP_003642924), CFD (XP_003642908), and PRSSS57 (XP_418216).

Zebra finch: MCP1-like (XP_002193027) and GzmE-like (XP_002193052).

Anolis: GzmA-like (XP_003216234), GzmA-like (XP_008100999), MCP-1A-like (XP_003228813), CtsG-like (XP_008120784),

Alligator: MCP1A-like (XP_006037527), MCP1-like (XP_006037528), MCP3-like (XP_006037530), MCP3-like (XP_006017535), DDN1-like (XP_006017536), CtsG-like (XP_006038557), CtsG-like (XP_006037787), GzmA (XP_006017354), GzmM-like (XP_006017616), CFD (XP_006017514) and PRSS57 (XP_006017629).

W.clawed frog: GzmH (NP_001238808), GzmA (NP_001107158), GzmA-like (XP_002941159), GzmA-like (XP_002941155), PRSS57 (XP_002937430), PRTN3 or NE (NP_001006847), CFD (NP_989320), PRSS57 (XP_002940708) and CtsG (NP_001107513).

Shark: GzmA-like (NP_001279383), GzmA-like (NP_001279379), GzmA-like (XP_007890376), and GzmK-like (AFK11031), GzmK-like (XP_007890378), GzmK-like (XP_007890379) and GzmK-like (NP_001279399).

Zebrafish; Arginine esterase like (XP_687163), Granzyme like protein-I-like (XP_003201101), Zebrafish UCP (NP_001076536), MCP1A (XP_009294635), DDN1 (XP_003201098), CtsG (XP_003201079), GzmG (XP_003201078), MCP1A (XP_003201097), DDN-1 (XP_003201096), Granzyme I-like (XP_003201077), Granzyme I-like (XP_005170582), UCP (NP_001038273), MCP1A (XP_003201076), MCP-3 (XP_003201094), Granzyme I-like (XP_003201075), DDN1 (XP_003201093), DDN-1 (XP_009294620), Granzyme I-like (XP_009294619) and CFD (NP_001018368).

Zebrambuna; GzmB-like (XP_004566138), GzmB-like (XP_004566122), GzmA-like (XP_004547390), GzmA-like (XP_004547009), GzmA-like (XP_004547010), GzmA-like (XP_004547011), GzmA-like (XP_004547391), GzmA-like (XP_004554973), CFD-like (XP_004554830), GzmG-like (XP_004554955), GzmG-like (XP_004554956), GzmB-like (XP_004554957), GzmB-like (XP_004554958), MCP1A-like (XP_004554960), GzmE-like (XP_004554959), Granzyme-I-like (XP_004554971), DDN1-like (XP_004555175), DDN1-like (XP_004554974), DDN1-like (XP_004554975), GranzymezmII-like (XP_004555177),

Nile tilapia; GzmA-like (XP_003455357), GzmK-like (XP_005459882), GzmA-like (XP_003455332), GzmG-like (XP_003439728) GzmG-like (XP_003439727), GzmB-like (XP_003439726), GzmB-like (XP_003439725), MCP2-like (XP_003439724), GzmE-like (XP_003439723), DDN1-like (XP_005479057), DDN1-like (XP_005479294), MCP1A-like (XP_003439903), GzmA-like (XP_003439712), DDN1-like (XP_003439710), MCP8-like (XP_005479059), GzmB-like (XP_003443311), GzmF-like (XP_005450435) and CFD-like (XP_003447819).

Astatotilapia buroni/haplochromic burtoni; GzmK-like (XP_005924587), GzmK-like (XP_005924539), GzmA-like (XP_004547010), GzmA-like (XP_005924541), CtsG-like (XP_005925944), GzmG-like (XP_005925942), CFD-like (XP_005929715), GzmB-like (XP_005920801), DDN1-like (XP_005920803), GzmA-like (XP_004554973), MCP2-like (XP-005920804), DDN1-like (XP_005920804), GzmII-like (XP_005920809), GzmBGH-like (XP_005920949), GzmB-like (XP_005920783) and GzmB-like (XP_005920784).

Medaka: GzmI-like (XP_004067875), DDN1-like (XP_004067877), MCP1A-like (XP_004067878), GzmE-like (XP_004086989) and GzmA-like (XP_004072307).

Fugu: MCP3-like (XP_003974113), GzmBGH-like (XP_003974114), DDN1-like (XP_003962119), GzmA-like (XP_003979723) and GzmK-like (XP_003975075).

Haplochromis Nyererei; GzmA-like (XP_005738215), GzmA-like (XP_005738216), GzmA-like (XP_005738217), GzmA-like (XP_005738218), GzmA-like (XP_005738219), GzmA-like (XP_005738334), GzmG-like (XP_005734067), GzmB-like (XP_005734071), GzmB-like (XP_005734070), GzmI-like (XP_005734073), GzmI-like (XP-005734088) and DDN1-like (XP_005734186).

GzmK-like (XP_005734091), DDN1-like (XP_005734187), DDN1-like (XP_005734092), GzmII-like (XP_005734188), GzmK-like (XP_005754665), MCP4-like (XP_005754662) and GzmBGH-like (XP_005754666).

Southern Platyfish; GzmI-like (XP_005795763), GzmE-like (XP_005795764), GzmBGH-like (XP_005795648), MCP8-like (XP_005795772), MCP3-like (XP_005795775), GzmA-like (XP_005798529), GzmA-like (XP_005798530), GzmB-like (XP_005816785) and GzmBGH-like (XP_005816786).

Spotted gar: CFD-like (XP_006640100), GzmG-like (XP_006640106), MCP2-like (XP_006640107), GzmG-like (XP_006640109), MCP1A-like (XP_006640110), CFD-like (XP_006640111), GzmA-like (XP_006626684) and GzmB-like (XP_006639143).

Pufferfish: GzmA (ENSTNIP00000019036), CMA1 (ENSTNIP00000013554), MCP1 (ENSTNIP00000013554), PRSS57 (ENSTNIP00000003419), MCP2 (ENSTNIP00000000945) and MCP3 (ENSTNIP00000013553).

Stickleback: GzmA (ENSGACP00000006218), CMA1 (ENSGACP00000009787), PRSS57 or AZU1 (ENSGACP00000017082), Sp1 (ENSGACP00000017093), Sp2 (ENSGACP00000017100) and sp3 (ENSGACP00000017104).

Ctfish: GrnzymeI-like (NP_001187138), GranzymeII-like (AAX36078) and GranzymeIII-like (NP_001187139).
